# Reversal of Chronic Stress‐Induced Inflammaging by Bazi Bushen Formula Through AMPK‐Sirtuins Axis Activation and Myeloid Reset

**DOI:** 10.1002/exp2.70235

**Published:** 2026-10-11

**Authors:** Mengnan Li, Zheming Chen, Lu Wang, Zixuan Wang, Shixiong Zhang, Kunxu Niu, Xiaogang Shen, Xuan Lu, Runtao Zhang, Yuning Jiang, Yawen Li, Yi Dai, Xiyang Tang, Bin Hou, Min Shi, Xiaoya Wang, Zhifang Guo, Xingyu Xing, Ning Kang, Hongrong Li, Tianyu Kang, Evelyne Bischof, Yunlong Hou, Zhenhua Jia

**Affiliations:** ^1^ State Key Laboratory For Innovation and Transformation of Luobing Theory Shijiazhuang China; ^2^ Hebei Provincial Administration of Traditional Chinese Medicine Anti‐Aging Research Key Laboratory Shijiazhuang China; ^3^ Hebei Medical University Shijiazhuang China; ^4^ LC‐BIO Technologies CO., LTD. Hangzhou China; ^5^ School of Chinese Medicine Nanjing University of Chinese Medicine Nanjing China; ^6^ State Key Laboratory of Bioactive Molecules and Druggability Assessment, Guangdong Basic Research Center of Excellence for Natural Bioactive Molecules and Discovery of Innovative Drugs, College of Pharmacy Jinan University Guangzhou PR China; ^7^ Sheba Longevity Center, Sheba Medical Center, Gray Faculty of Medical and Health Sciences Tel Aviv University Ramat Gan Israel; ^8^ Renji Hospital of the Jiaotong University School of Medicine, Department of Oncology and Clinical Cancer Center, Department of Oncology Shanghai Jiao Tong University Shanghai China; ^9^ Medicine Department Shanghai University of Medicine and Health Sciences Shanghai China; ^10^ Key Laboratory of State Administration of TCM (Cardio‐Cerebral Vessel Collateral Disease) Shijiazhuang China; ^11^ Hebei Academy of Integrated Traditional Chinese and Western Medicine Shijiazhuang China

**Keywords:** AMPK‐SIRT‐PGC‐1α pathway, lymphocyte‐myeloid imbalance, multicomponent pharmacological agents, multi‐organ inflammaging, unpredictable chronic mild stress

## Abstract

Chronic stress potentially accelerates aging and increases age‐related disease risk. However, the underlying mechanisms and organ specificity remain unclear. Utilizing a 12‐month unpredictable chronic mild stress (UCMS) mouse model, we revealed chronic stress significantly exacerbates systemic inflammation, causing widespread tissue damage and lifespan shortening. Integrated single‐cell RNA sequencing analysis of multiple tissues suggests a common feature of lymphocyte reduction and myeloid cell expansion. The lungs and kidneys exhibited pronounced stress responses, including epigenetic dysregulation and mitochondrial dysfunction. Both β‐nicotinamide mononucleotide (NMN) and compound medicine Bazi Bushen (BZBS) effectively alleviate the UCMS‐induced aging phenotypes. BZBS restores immune system homeostasis and reduces stress/inflammation levels in various tissues. Bioactive compounds from BZBS constituents*—Schisandra chinensis (Turcz.) Baill. and Cnidium monnieri (L.) Cusson—*improved mitochondrial function via AMPK‐SIRT‐PGC‐1α signaling activation. The present study underscores the importance of early intervention in chronic stress to prevent or delay age‐related diseases, with significant implications for public health strategies aimed at healthy aging.

## Graphical abstract

Chronic stressors trigger systemic immune remodeling marked by myeloid expansion and lymphoid suppression, and this immune imbalance subsequently drives tissue pathology through proinflammatory signaling and senescence amplification, with the lung and kidney confirmed as the most vulnerable target organs. These cascading pathological alterations further accelerate the decline of healthspan and the shortening of overall lifespan in the organism. Bazi Bushen (BZBS) can effectively counteract the above adverse effects through a synergistic regulatory mechanism that precisely targets mitochondrial dysfunction, genomic instability, and epigenetic dysregulation.

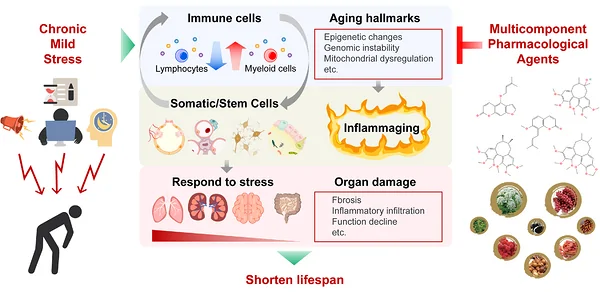

## 1 Introduction

Chronic stress, characterized by sustained physiological and psychological strain due to demanding social environments, is increasingly prevalent in today's fast‐paced society. Accumulating evidence indicates that chronic stress extends beyond impairing mental well‐being; it not only elevates risks for age‐related diseases (e.g., type 2 diabetes, Alzheimer's disease, and cancers [1, 2, 3, 4]) but also accelerates epigenetic aging, as demonstrated by recent human cohort studies linking prolonged stress to DNA methylation aging clocks and immune senescence markers [5, 6, 7]. Although the causative relationship and mechanism between the intensity and duration of chronic stress and aging phenotypes remain to be fully elucidated, current evidence implicates stress‐induced hypothalamic‐pituitary‐adrenal (HPA) axis dysregulation, autonomic nervous system (ANS) imbalance, telomere attrition, and oxidative stress accumulation in driving premature aging phenotypes [8, 9, 10, 11].

Recent investigations have also highlighted the potential interaction between chronic stress and inflammation, suggesting that long‐term exposure to adverse conditions can lead to low‐grade inflammation. Indeed, elevated cortisol disrupts immune function, releasing pro‐inflammatory cytokines and activating immune cells under chronic stress [12]; telomere shortening, potentially accelerated by stress, can contribute to inflammation as damaged cells emit inflammatory signals [13, 14]; oxidative stress damages DNA, proteins, and lipids, all of which can further promote inflammation [15]. Critically, emerging clinical studies reveal that chronic stress remodels immune profiles in human populations, with elevated pro‐inflammatory cytokines (e.g., IL‐6, TNF‐α) and depleted naïve T‐cell subsets mirroring aged immune systems [7, 16]. These shifts align with the “inflammaging” paradigm—a self‐reinforcing cycle of sterile inflammation that exacerbates tissue degeneration [17]. Notably, stress‐associated epigenetic changes (e.g., glucocorticoid receptor methylation) may potentiate inflammaging by disrupting immune cell homeostasis [18, 19]. However, key gaps persist in understanding how stress differentially reprograms immune and epigenetic networks across organs and whether these effects are reversible.

Another question worth investigating is whether pharmacological interventions can mitigate chronic stress‐induced aging phenotypes. Emerging anti‐aging candidates include NMN, flavonoids, metformin, etc., many of which lifespan‐extending compounds validated in natural aging models of mammals are derived from phytochemical sources. Representative examples include fisetin—a dietary polyphenol abundant in strawberries and persimmons, luteolin from *Salvia officinalis L*., and extracts from *Salsola collina Pall*.‐derived extract JM10001 [20, 21, 22]. Bazi Bushen (BZBS)—a patent compound Chinese medicine—merits particular attention in this context. This formulation integrates eight medicinal plant seeds and longevity herbs, including *Schisandra chinensis (Turcz.) Baill*., *Cnidium monnieri (L.) Cusson*, *Lycium barbarum L*., *Ginseng Radix et Rhizoma (Panax ginseng C.A.Mey.)*, and *Cistanches Herba (Cistanche deserticola Y.C.Ma)*. Accumulating evidence demonstrates BZBS's broad anti‐aging properties: preclinical studies show it extends healthspan in naturally aged mice while improving cognitive function, musculoskeletal integrity, and reproductive hormone homeostasis [23, 24, 25, 26]. Given the necessity for long‐term therapeutic regimens in stress stimulation, plant‐derived agents that exhibit dual functionality (dietary compatibility and pharmacological activity) present potential safety and sustainability advantages. Furthermore, the multifactorial nature of aging mechanisms suggests that polypharmacological approaches may yield superior outcomes by concurrently targeting multiple pathways. However, the efficacy of BZBS in reversing stress‐induced functional deterioration and the molecular identities of its active constituents remain unknown.

In this study, we employed a translational UCMS‐incorporating noise disturbance, circadian rhythm disruption, and suboptimal living conditions to model modern societal stressors in 11‐month‐old C57BL/6 mice. The UCMS model effectively mimics human chronic stress by integrating unpredictable psychosocial and physiological stressors, progressively inducing anhedonia, metabolic dysregulation, and HPA axis hyperactivity similar to long‐term human stress exposure [27, 28, 29]. The mouse age was strategically selected as it corresponds to human middle age (∼40‐45 years) [30, 31] and exhibits minimal baseline aging phenotypes under standard conditions [30, 31], allowing clear isolation of stress‐accelerated effects. Through integrated behavioral tests, pathological evaluations, and multi‐organ single‐cell sequencing, we systematically characterized both shared and tissue‐specific manifestations of stress‐accelerated aging while evaluating the protective potential of prophylactic compound medicine administration.

## 2 Materials and Methods

### 2.1 Long‐Term Chronic Stress Modeling and Drug Administration

One hundred and twenty‐five females and 126 males of C57BL/6 mice (11 months old), along with 6 young male controls (4 months old), all of specific pathogen‐free grade, were obtained from Beijing Huafukang Biotech Co., Ltd., China. The mice were housed in a controlled lab environment with a temperature of 25 ± 2°C, relative humidity of 60%–70%, and a 12‐hour light‐dark cycle. They had ad libitum access to food and water. After a one‐week acclimation period, the mice were randomly assigned to five groups: Control, Model, NMN (200 mg/kg/d), high dose of BZBS (2 g/kg/d, BZ‐high), and low dose of BZBS (1 g/kg/d, BZ‐low). The doses of NMN (Lot: BT05N120A005, Bontac Bio‐engineering Co. Ltd., China) and BZBS (Lot: SYB2112001, Yiling Pharmaceutical Co., Ltd., China) were selected based on the protocol established in our prior research [32]. The medicinal herbal ingredients of BZBS are listed in Supplementary Table S1. Chronic mild stress modeling was carried out using ten methods rotating daily [33, 34], including cage tilting, isolation, crowding, damp environment exposure, rapid light‐dark cycling, nocturnal illumination, white noise exposure, continuous light or darkness exposure, and water deprivation. Notably, intermittent fasting was excluded from the study due to potential anti‐aging effects [35]. After 12 months of drug administration, mice underwent biomolecular testing and pathological examination. Chemical analysis of BZBS was performed by ultra‐performance liquid chromatography/mass spectrometry (UPLC/MS) [36].

### 2.2 Three‐Chambered Social Test

The test setup consisted of three boxes separated by transparent glass resin panels. Test mice were initially placed in the middle box for a 5‐minute adaptation period. A new mouse, Stranger1, was then introduced randomly into one of the side metal cages. After removing the panels, the mice were allowed to freely explore the three boxes for 10 min. The interaction of test mice with Stranger1 and the empty cage was recorded. Subsequently, another unfamiliar mouse, Stranger2, was introduced into the remaining empty cage, and interactions between the test mice and both Strangers were monitored for 10 min.

### 2.3 Barnes Maze Test

The Barnes maze (Shanghai JiLiang, Shanghai, China, JLBehv‐BNSG) was used to assess spatial ability/memory. The platform consists of an 80 cm wide circular maze with 20 holes. Before the formal test, mice underwent training, which allowed them to explore the maze freely for 180 s until they entered the escape hole. Training sessions were conducted twice per day for 3 consecutive days before the formal test. Each test was recorded by a camera installed above the platform, and videos were analyzed using DigBehv software (Shanghai JiLiang, Shanghai, China, JLBehv‐BNSG).

### 2.4 Grip Test and Rotarod Test

The mice were placed on the grip strength tester (Columbus Instruments Co., Ltd). By gently drawing the mice backwards, their grip strength values were recorded upon releasing from the grid. The highest value from three tests was retained. Muscle endurance was assessed using the IITC Life Sciences rotarod test with specific parameters. Mice underwent three trials with 30‐minute intervals, and their time on the rotating rod was measured to calculate total time and distance based on the mean of the three trials.

### 2.5 Mouse Frailty Assessment

Frailty index (FI) is a widely utilized method for evaluating physiological conditions of mice [37]. The mouse frailty index (FI) was assessed 1–2 months before endpoint. For more details, see http://frailtyclocks.sinclairlab.org/. Briefly, the weight and core temperature of the mice were measured weekly. The rest of the assessments were performed at the end of treatment and included assessments of (1) alopecia; (2) fur color loss; (3) dermatitis; (4) coat condition; (5) loss of whiskers; (6) kyphosis; (7) distended abdomen; (8) vestibular disturbance; (9) cataract/corneal opacity; (10) eye discharge/swelling; (11) microphthalmia; (12) malocclusions; (13) rectal prolapse; (14) penile prolapse; (15) mouse grimace scale; (16) piloerection; (17) tail stiffening; (18) gait; (19) grip strength; (20) body condition; (21) hearing loss; (22) vision loss; (23) menace reflex; (24) tremor; (25) tumors or lumps; (26) nasal discharge; (27) diarrhea; and (28) breathing rate/depth. We scored each item from 0 to 1 based on the severity. The average value of all 30 items represents the FI score.

### 2.6 Nuclear Magnetic Resonance Imaging (MRI) of Mouse Brain

Three mice from each group were chosen randomly for MRI scanning using the Bruker 500 MHz 11.7T system from Germany. The mice were anesthetized with 1.5% isoflurane and oxygen, monitored for stable vital signs, and positioned using an animal respirator. T2‐weighted images were obtained over a 6‐minute and 24‐second scan with the following parameters: TR = 3500 ms, TE = 30 ms, fov = matrix, and a total scan time of 6 min and 24 s. Brain region volumetric data were acquired based on a mouse brain template in Paxinos space, involving spatial alignment and transformation processes to generate regionally segmented images in the individual space.

### 2.7 Histopathological Analysis

Tissue was fixed in 4% paraformaldehyde, embedded in paraffin, sectioned, and stained with hematoxylin and eosin (H&E) and Masson's trichrome. Histological analysis was performed with a light microscope. Inflammation and fibroplasia were scored based on the accepted criteria and the modified histological activity index (HAI) [38]. The tissue sections of each group were scored according to the HAI by two expert pathologists.

### 2.8 RNA Isolation and Quantitative Real‐Time PCR

Total RNA was extracted using the TransZol Up Plus RNA Kit (TransGen Biotech, Beijing, China, ER501‐01) and reverse transcribed with the GoScript Reverse Transcription System (Promega, Madison, USA, A5001). The cDNA products were then used as templates for qPCR with the MonAmp ChemoHS qPCR Mix kit (Monad, Suzhou, China, MQ00401S). The primer sequences are listed in Supplementary Table S2. All experiments followed the manufacturer's instructions.

### 2.9 Immunofluorescence

Tissues were embedded in Sakura Tissue‐Tek OCT Compound (Sakura, USA, 4583) and sectioned into slices of 8 µm thickness. Tissue slices were then fixed in 4% paraformaldehyde for 10 min. Blocking of the liver sections was conducted with Triton X‐100 (Solarbio, China, T8220) for 60 min. Slides were incubated with primary antibody (Supplementary Table S3) at 4°C overnight, and the secondary antibody (Supplementary Table S3) was added for 40 min in the dark. DAPI (SouthernBiotech, 0100–20) was used for nuclear staining. High‐resolution images were acquired using a confocal microscope (Zeiss, Germany, LSM710) and were analyzed with ImageJ.

### 2.10 Oxidative Stress Measurement

The levels of protein carbonyl (PCO) and malondialdehyde (MDA) were measured using assay kits obtained from Suzhou Grace Biotechnology Co., Ltd. (Cat. No.: G0130W; G0109W). The level of Glutathione (GSH) and the activity of superoxide dismutase (SOD) were measured using assay kits obtained from Solarbio (Beijing, China) and Biyuntian (Shanghai, China). All operations followed the manufacturer's instructions.

### 2.11 Reduced Representation Bisulfite Sequencing (RRBS) Analysis and Epigenetic Aging Clock Analysis

Total DNA was extracted using a QIAamp Fast DNA Tissue Kit (Qiagen, Dusseldorf, Germany) following the manufacturer's procedure. The quantity of DNA was measured by reading the A260/280 ratios with a spectrophotometer. When the A260/280 ratios ranged from 1.8 to 2.0, it indicated that DNA was available. The DNA samples were fragmented by using the restriction endonuclease Mspl (NEB, USA) and then were subjected to bisulfite conversion, The Accel‐NGS Methyl‐Seq DNA Library Kit (Swift, MI, USA) was utilized to attach adapters to single‐stranded DNA fragments. Briefly, the following protocol was used. First, the Adaptase step was performed, with Adaptase being a highly efficient, proprietary reaction that simultaneously performs end repair, tailing of 3' ends, and ligation of the first truncated adapter complement to 3’ends. Then, the extension step was used to incorporate truncated adapter 1 by means of a primer extension reaction. This was followed by the ligation step, which was used to add the second truncated adapter to the bottom strand only. The indexing PCR step was then used to increase yield and incorporate full‐length adapters. Next, bead‐based solid‐phase reversible immobilization (SPRl) clean‐ups were used to remove both oligonucleotides and small fragments, as well as to change the enzymatic buffer composition. Finally, we performed paired‐end 2 × 150 bp sequencing on an Illumina HiSeq 4000 platform housed in LC Sciences. The DNA methylation age was calculated as previously described [23].

### 2.12 Immunoblotting

Total protein was extracted from cells and tissues using RIPA lysis buffer (Seven, SW104‐01, China). Protein concentrations were determined by BCA assay (Seven, SW101‐02, China). Equal amounts of protein were separated by SDS‐PAGE and transferred to nitrocellulose membranes, which were blocked at room temperature for 1 h. Membranes were incubated overnight at 4°C with primary antibodies (Supplementary Table S3), followed by 1 h incubation with HRP‐conjugated secondary antibodies at room temperature. Protein bands were visualized by chemiluminescence and imaged on a Western blot imaging system. Densitometric analysis was performed using ImageJ software (NlH, USA).

### 2.13 Single‐Cell RNA Sequencing

For clustering and identifying cell types in single tissue, we enhanced resolution across cell types in each tissue by performing single‐cell RNA sequencing (scRNA‐seq) data analysis following Steps 1–5 to create a single‐cell atlas for each tissue. The Cell Ranger was used to calculate single‐cell expression matrices, and the Seurat package (version 4.0) was utilized for filtering, normalizing data, reducing dimensionality, clustering, and analyzing gene differential expressions. Various steps were taken: excluding cells with less than 500 detected genes from all scRNA‐seq data, removing cells with a mitochondrial gene ratio exceeding 25% in samples, detecting and filtering doublets with the DoubletFinder package (version 2.0.3), normalizing data, identifying the 2000 most variable genes, constructing a shared‐nearest‐neighbors graph, and clustering cells using the UMAP algorithm followed by assigning cell type annotations based on canonical marker genes with further analyses conducted on these cell types among the five sample types.

The analysis included differential expressions and cell type‐specific DEG networks. The Wilcoxon rank‐sum test in the Seurat package was used to analyze differential expressions between cell types in different groups. Cell types with fewer than ten cells were filtered out before conducting the analysis. DEGs were identified based on certain criteria. An integrated analysis of B cell expression profiles from five organs was conducted, based on the methodology introduced by Mathew et al. [39] and utilizing the Seurat package and excluding irrelevant genes. Batch effect mitigation and data integration were performed using Harmony. Trajectory inference analysis of B cells was carried out using Monocle 3 and CellRank for lineage trajectory exploration and validation.

The study used gene set expression analysis to examine aging‐related gene sets acquired from Kyoto Encyclopedia of Genes and Genomes (KEGG), Gene Ontology (GO), and Human Ageing Genomic Resources (HAGR) (https://genomics.senescence.info/). Heatmaps and gene expression profiles were generated using the pheatmap R package. For gene ontology analysis, Metascape was used for GO enrichment, with results visualized using Cytoscape or ggplot2. Cell‐cell communication was analyzed with CellPhoneDB, evaluating only receptors and ligands expressed in at least 10% of cells. Statistical analysis included t‐tests and Wilcoxon tests, with a significance level of *p* < 0.05. The aging hallmarks gene set was scored using Seurat's AddModuleScore, and the gene set is shown in Supplementary Table S4. The siAge scoring was based on the previous work of Wang et al. [40].

### 2.14 Pharmacokinetics

BZBS capsule content was dissolved in sterile water at doses of 0.2 g/mL for gavage. BZBS was orally administrated to 8 rats (BZBS group) at dosages of 1 g/kg/day for three days, and the rest 2 rats (blank group) were given an equal amount of sterile water, respectively. Whole blood samples were collected at 0.5 h, 1 h, 2 h and 4 h (four time points) after the last administration. Blood samples were centrifuged at 13,200 rpm (10°C) for 10 min, and plasma was separated into a clean tube and stored at −80°C until drug analysis. The blank plasma sample was taken by the same method from the rats in the blank group.

The analysis was running on a separation system: ACQUITY UPLC I‐Class system equipped with a binary solvent system and an automatic sample manager that was equipped with an ACQUITY UPLC BEH C18 column (2.1 mm×100 mm, 1.7 µm) at a temperature of 35°C. The mobile phases consisted of eluent A (0.1 % formic acid in water, v/v) and eluent B (0.1 % formic acid in acetonitrile, v/v). These phases were delivered at a flow rate of 0.4 mL/min by using a linear gradient program as follows: 0–3.5 min, 2%‐15% B; 3.5‐12.5 min, 15%‐36% B; 12.5‐19.5 min, 36%‐55% B; 19.5‐23.5 min, 55%‐80% B; 23.5‐26 min, 80%‐100% B; 26–28 min, 100% B; 28–29 min, 100%‐2% B; 29–30 min, 2% B. The injection volume of all samples was 2 µL.

The UPLC system was coupled with a quadrupole time‐of‐flight tandem mass spectrometer (SYNAPT G2 HDMS; Waters, Manchester, U.K.) equipped with an electrospray ionization (ESI) source. The operating parameters in MSE mode were set as follows: capillary voltage of 3.0 kV (ESI+); sample cone voltage of 35 V in positive mode and 40 V in negative mode; source temperature of 100°C; desolvation temperature of 300°C; cone gas flow of 50 L/h; and desolvation gas flow of 800 L/h. In MSE mode, the trap collision energy of the low‐energy function was set at 5 eV, while the ramp trap collision energy of the high‐energy function was set at 20−50 eV. To ensure mass accuracy and reproducibility, the mass spectrometer was calibrated over a range of 50−1500 Da using sodium formate solution. Leucine‐enkephalin (m/z 556.2771 in positive ion mode) was used as the external reference of LockSpray infused at a constant flow of 5 µL/min. Data acquisition was performed by Masslynx 4.1 software (Waters, Manchester, U.K.) in positive and negative ion modes.

### 2.15 Network Pharmacology

Construction of compound targets‐oxidative stress network: The targets of active compounds were obtained from DrugBank, TTD, ChEMBL, and PubChem and standardized their names through UniProt. Using “Oxidative Stress” as the search term, the oxidative stress gene was retrieved from five sources, including DisGeNET, Open Target Platform, MalaCards, CTD, and GeneCards. The target protein name was converted to the official symbol formats for gene targets using UniProt (https://www.uniprot.org/).

Analysis of protein‐protein interaction (PPI) network and function enrichment: The intersection targets between BZBS and oxidative stress were obtained and visualized with a Venn diagram by Wei Sheng Xin (https://www.bioinformatics.com.cn/). Targets of genes at the intersection were imported into STRING, and the confidence score was set to ≥0.4 (http://string‐db.org). Cytoscape 3.10.2 was used to construct a network of potential key targets and to perform a systematic analysis of the network parameters. Intersecting genes were evaluated by GO and KEGG enrichment analysis via Wei Sheng Xin, with FDR < 0.05 and *p* < 0.05.

### 2.16 Virtual Docking

Molecular docking method was used to virtually evaluate the binding affinity between the compound with target proteins, which might provide a reference for further experimental verification. AutoDock Vina 1.1.2 is an open‐source program for molecular docking and virtual screening. Compared with AutoDock 4, its average accuracy of binding mode prediction is much higher. Compounds of SDF files downloaded from the PubChem database (https://pubchem.ncbi.nlm.nih.gov/) and through the Open Babel against 2.4.1 into MOL2 format. The crystal structures of AMPK (PDB ID: 5EZV), Sirtuin 1 (PDB ID: 4ZZH), Sirtuin 3 (PDB ID: 4BN4), and Sirtuin 6 (PDB ID: 6XV1) were downloaded from PDB database (https://www.rcsb.org/). Small molecules were energy‐minimized by using PyRx‐0.8 software. Ligands and receptors were prepared according to the AutoDock Vina 1.1.2 tutorial. For each structure, we removed water molecules, added nonpolar hydrogen, calculated Gasteiger charges, and saved them in the PDBQT format. Generally, the lower the Vina score, the higher the Affinity between ligand and receptor. The general selection threshold is binding energy ≤ −5 kcal/mol. Finally, Pymol software was used to visualize the interaction mode between the compound and target proteins.

### 2.17 Cell Culture

Rat Brain Astrocytes Cells CTX were purchased from CytoKin (Cat. No.: iCell‐r007). Mouse mononuclear macrophage leukemia cells Raw264.7 (Cat. No.: ZQ00981) and special culture medium (Cat. No.: ZM0098) were purchased from Zhongqiao Xinzhou. Cells were cultured in a humidified atmosphere with 5% CO_2_ at 37°C. The reagents used in the cell experiments are listed in Supplementary Table S5.

### 2.18 Astrocyte Viability Assessment

Rat astrocytes were seeded in 96‐well plates (1 × 10^5^ cells/mL, 100 µL/well) and cultured for 24 h prior to compound exposure. Cell viability was quantified using MTS assays 24 h post‐treatment with graded concentrations of the test compound.

### 2.19 Mitochondrial Superoxide Detection

For H_2_O_2_ challenge: Following 24 h culture in 96‐well plates (1 × 10^5^ cells/mL), cells were pre‐treated with test compounds for 24 h prior to oxidative stress induction. Cells were incubated with HBSS (Ca^2+^/Mg^2+^ supplemented) containing 500 µM H_2_O_2_ and 500 nM MitoSOX Red probe (100 µL/well) at 37°C. Fluorescence intensity was quantified using a microplate reader after 30 min incubation.

For CCCP challenge: The experimental timeline paralleled H_2_O_2_ treatment, substituting 50 µg/mL CCCP as the mitochondrial stress inducer. Real‐time imaging or plate reader detection followed identical instrumentation parameters.

### 2.20 Mitochondrial Membrane Potential Assessment

CTXs seeded in 24‐well plates (2.5 × 10^5^ cells/mL, 250 µL/well) underwent compound pre‐treatment for 24 h before exposure to H_2_O_2_ (500 µM) or CCCP (50 µg/mL) for 30 min at 37°C. JC‐1 staining was performed according to the manufacturer's protocol, with subsequent quantification via microplate reader, live‐cell imaging system, or confocal microscopy.

### 2.21 Seahorse Assay

Using XF24 Seahorse plates (CTXs, 1×10^5^ cells/mL, 100 µL/well), CCCP‐challenged astrocytes (50 µg/mL, 30 min) were analyzed for mitochondrial bioenergetics following standardized protocols. Oxygen consumption rates (OCR) and extracellular acidification rates (ECAR) were measured under basal and compound‐treated conditions.

### 2.22 Functional Comparison of BZBS With Anti‐Aging Compounds

The RAW264.7 cells (1 × 10^5^ cells/3.5 cm dish) were pretreated with BZBS (100 µg/mL), NMN (500 µM) [41, 42], or ABT‐263 (5 µM) [43, 44] or vehicle control for 3 days and then underwent irradiation (Rad Source Technologies Inc., USA, RS2000 X‐ray irradiator) at a dose of 6 Gy for 5 min. After irradiation, cells were cultured at 37°C, 5% CO_2_ for 5 days, with the culture medium changed every two days. 10 days post‐irradiation, cell state was assessed. The normal control group received no irradiation or drug treatment, while the irradiated control group was exposed to radiation but received no drugs.

### 2.23 AMPK Kinase Activity Assay

Rat astrocytes (5 × 10^6^ cells/15 cm dish) were subjected to CCCP‐induced oxidative stress (50 µg/ml, 30 min at 37°C) combined with pharmacological intervention. Following 24 h culture, cells received co‐treatment with the test compound and 10 µM Compound C (AMPK inhibitor) for 24 h prior to mitochondrial stress induction. AMPK enzymatic activity was quantified via spectrophotometric kinase activity assays following manufacturer protocols.

### 2.24 DNA Damage Quantification

RAW264.7 were seeded in 96‐well plates (1 × 10^5^ cells/ml, 100 µl/well). At 80% confluency, cells were pretreated with test compounds followed by 6 Gy γ‐irradiation. Post‐irradiation incubations proceeded for 24 h prior to fixation and γH2AX foci analysis via immunofluorescence microscopy. Radiation controls (3 wells/group) received equivalent sham treatments.

### 2.25 Biolayer Interferometry (BLI)

The binding affinities between compounds and target proteins were evaluated with the BLI‐based Octet Platform (Octet Red 96, Fortebio, USA). Assay buffer consisting of 1× phosphate buffered saline (PBS) (pH = 7.4) containing 0.04% Tween‐20 and 0.1% DMSO. Purified His‐tagged AMPKα1 (IPODIX, PA2000‐534DB, 50 µg/ml), AMPKα2 (elyue, FY‐P522491, 50 µg/ml), SIRT1 (Sino Biological, 11748‐H07E, 50 µg/ml), SIRT3 (Abcam, ab97951, 50 µg/ml), and SIRT6 (Sino Biological, S40‐31H, 50 µg/ml) were attached to Ni‐NTA biosensors (18‐5101, ForteBio, USA) through a sequence of equilibration, coupling, and a second equilibration, achieving a saturation response of 4–6 nm. To determine the binding kinetics, the interaction between AMPK, SIRT6, and compounds was separately measured over 90 s for association and another 90 s for dissociation. To correct the baseline shift, reference wells with just buffer solution were included. Another Ni‐NTA control sensor was set up, which was incubated solely in the buffer and prepared as a negative reference control to correct for any non‐specific binding of the compounds to the biosensor surface. A double reference subtraction approach, which involved subtracting both the background and nonspecific binding signals, was utilized to analyze the raw kinetic data. The binding affinity constant *K*_D_ (where *K*_D_ = *k*_dis_/*k*_on_, with *k*_on_ represent the association rate constant and *k*_dis_ representing the dissociation rate constant) values were determined with a 1:1 binding model through global fitting of multiple kinetic traces.

### 2.26 Cellular Thermal Shift Assay (CETSA)

To confirm that the BZBS‐series compounds specifically target AMPK, SIRT1, SIRT3, and SIRT6, we performed CETSA‐WB (Cellular Thermal Shift Assay coupled with Western Blotting) experiments. Following 24‐hour treatment with BZBS‐series compounds, HEK293T cells were washed twice with ice‐cold PBS buffer. Cell lysis was then performed using RIPA lysis buffer (containing protease and phosphatase inhibitor cocktails). The lysates were centrifuged at 15,000×g for 20 min at 4°C. The extracted protein samples were equally divided into six aliquots and subjected to heating at 37°C to 82°C for 3 min each, followed by incubation at 4°C for 3 min. The supernatants were mixed with loading buffer and heat‐denatured at 100°C for 7 min in microcentrifuge tubes. Finally, target proteins were quantitatively analyzed by Western blotting.

### 2.27 SiRNA‐Mediated AMPKα1 and AMPKα2 Knockdown

For AMPKα1 and AMPKα2 knockdown, CTX and RAW264.7 cells were seeded in 6‐well plates and transfected at 60–70% confluency using Lipofectamine 3000 with specific siRNA or negative control siRNA (GenScript Biotech Co., Ltd. Nanjing Branch, Supplementary Table S2). After 6 h transfection, the complete medium was replaced, and cells were cultured for an additional 48 h. Knockdown efficiency was confirmed by qRT‐PCR and western blot before functional assays.

### 2.28 Statistical Methods and Text Refinement

All statistical analyses were performed using IBM SPSS 22. Data were checked for normal distribution (Shapiro–Wilk test) and homogeneity of variance (Levene's test for equality of variance) before choosing the appropriate statistical test. For normally distributed data, one‐way analysis of variance (ANOVA) was used for multi‐group comparisons, followed by independent two‐sample Student's *t*‐test for comparisons between two groups. The Kruskal–Wallis test was used for nonnormally distributed data analysis. P values below 0.05 were considered significant for all analyses. All data are presented as the mean ± standard deviation (SD) or ± standard error of measurement (SEM).

## 3 Results

### 3.1 Chronic Stress Accelerates Multisystem Senescence and Lifespan Decline Ameliorated by Anti‐Aging Therapies

C57BL6 mice were subjected to 10 different mild stressors over a 10‐ to 12‐month period (Figure 1a). Physiological validation confirmed successful UCMS induction, as stressed mice exhibited elevated serum aldosterone levels and body weight loss compared to controls (Supplementary Figure S1a,b). Notably, at baseline (pre‐stress), 11‐month‐old mice showed no significant aging‐related decline in FI, rotarod performance, grip strength, or Barnes maze performance compared to 4‐month‐old young controls (Supplementary Figure S1c–g), suggesting that spontaneous aging phenotypes were minimal at this stage. Aligning with prior research on UCMS [33, 34], mice exposed to chronic stress without dietary restrictions exhibited a decrease in social novelty preference revealed by a three‐chamber social interaction test (Figure 1b,c). Furthermore, the Barnes maze test demonstrated that the spatial memory ability of stressed mice is significantly reduced (Figure 1d–f). The administration of distinct anti‐aging drugs ameliorated their cognitive decline to varying extents (Figure 1b–f). Aside from the central nervous system, stressed mice exhibited significant reductions in rotarod performance and grip strength compared to the control mice, while drug‐treated stressed mice showed improved motor functions (Figure 1g,h). Notably, mice subjected to chronic stress stimulation exhibited overall significantly higher FI values compared to the Control group and drug‐treated groups in both male and female mice (Figure 1i,j and Supplementary Table S6), especially in hair whitening, shedding, vestibular dysfunction, and diarrhea phenotypes (Figure 1k–n). These findings all suggested a reduction in the healthy lifespan of mice due to chronic stress. More surprisingly, upon prolonged stimulation, stressed mice possessed a significantly elevated mortality rate. Both drug treatments exhibited varying degrees of lifespan extension among stressed mice (Figure 1o). Notably, High‐dose BZBS outperformed NMN in decreasing the FI, the death rate, and extending lifespan (Figure 1i–p).

**FIGURE 1:**
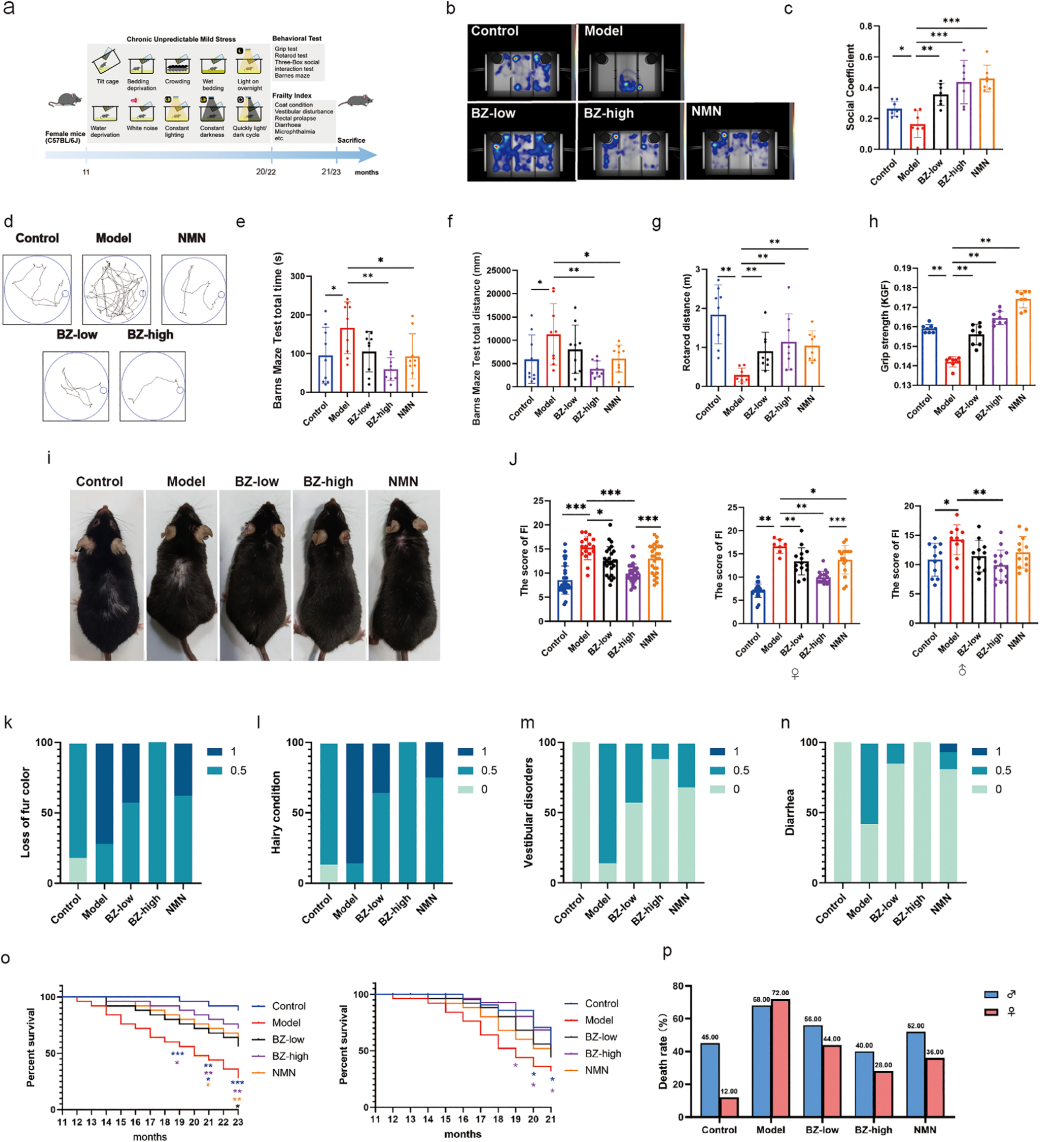
Chronic stress accelerates multidimensional aging trajectories partially reversed by therapeutic interventions. (a) Experimental timeline of 12‐month unpredictable chronic mild stress (UCMS) exposure with therapeutic administration. (b–c) Three‐chamber social interaction test showing reduced social novelty preference in stressed mice (*n* = 7). (d–f) Barnes maze analyses demonstrating spatial memory deficits and recovery with treatments (*n* = 9). (g–h) Motor function impairments and drug‐mediated partial improvements (*n* = 8). (i) Stress‐induced aging phenotypes. (j) Elevated Frailty Index scores in total (left), female (middle) (*n* = 7‐22) and male (right) (*n* = 10‐15) stressed cohorts. (k–n) Hair whitening, shedding, vestibular dysfunction, and diarrhea phenotypes scoring in each group of mice. (o) Survival curves showing accelerated mortality and protective effects of treatments female (left) (*n* = 25) male (right) (*n* = 20‐25). (p) Death rate in each group of mice. Data presented as mean ± SD. Statistical significance determined by one‐way ANOVA with Tukey post hoc (multi‐group, ***p < 0.001, **p < 0.01, and *p < 0.05).

Further pathological tests were performed on various tissues of the mice. MRI results demonstrated no notable variations in brain volume across groups, while stressed mice exhibited significant atrophy in the white matter region (Figure 2a–c). Additionally, the lengths of the intestine and proportions of muscle and fat of stressed mice were remarkably reduced (Figure 2d–j). All drug treatment groups showed varying degrees of relief in tissue degenerations, except for NMN, which failed to effectively reverse the fat proportion (Figure 2a–j). H&E and Masson staining demonstrated excessive keratinization, thicker epidermis, and disorganized collagen fibers (Figure 2k(i),l and Supplementary Figure S2a,b). Ileum pathology results demonstrated mucosal atrophy in the Model group, marked by sparse villi, reduced length, and shortened colon length (Figure 2k(ii), m). H&E staining of muscle tissue (Figure 2k(iii),n) and adipose (Figure 2k(iv)) revealed disordered and loosely packed muscle fibers and adipocyte atrophy in the Model group. Renal pathology revealed that long‐term chronic stress resulted in obvious tubular dilation and tubular degeneration (Supplementary Figure S1c). The heart and lungs also showed varying degrees of severe structural damage (Figure 2k(v) and Supplementary Figure S2d). In addition, inflammatory cell infiltration was also observed in multiple tissues (Figure 2k and Supplementary Figure S2). Of note, administration of BZBS and NMN attenuated the UCMS‐induced structural abnormalities across multiple organs (Figure 2k and Supplementary Figure S2).

**FIGURE 2:**
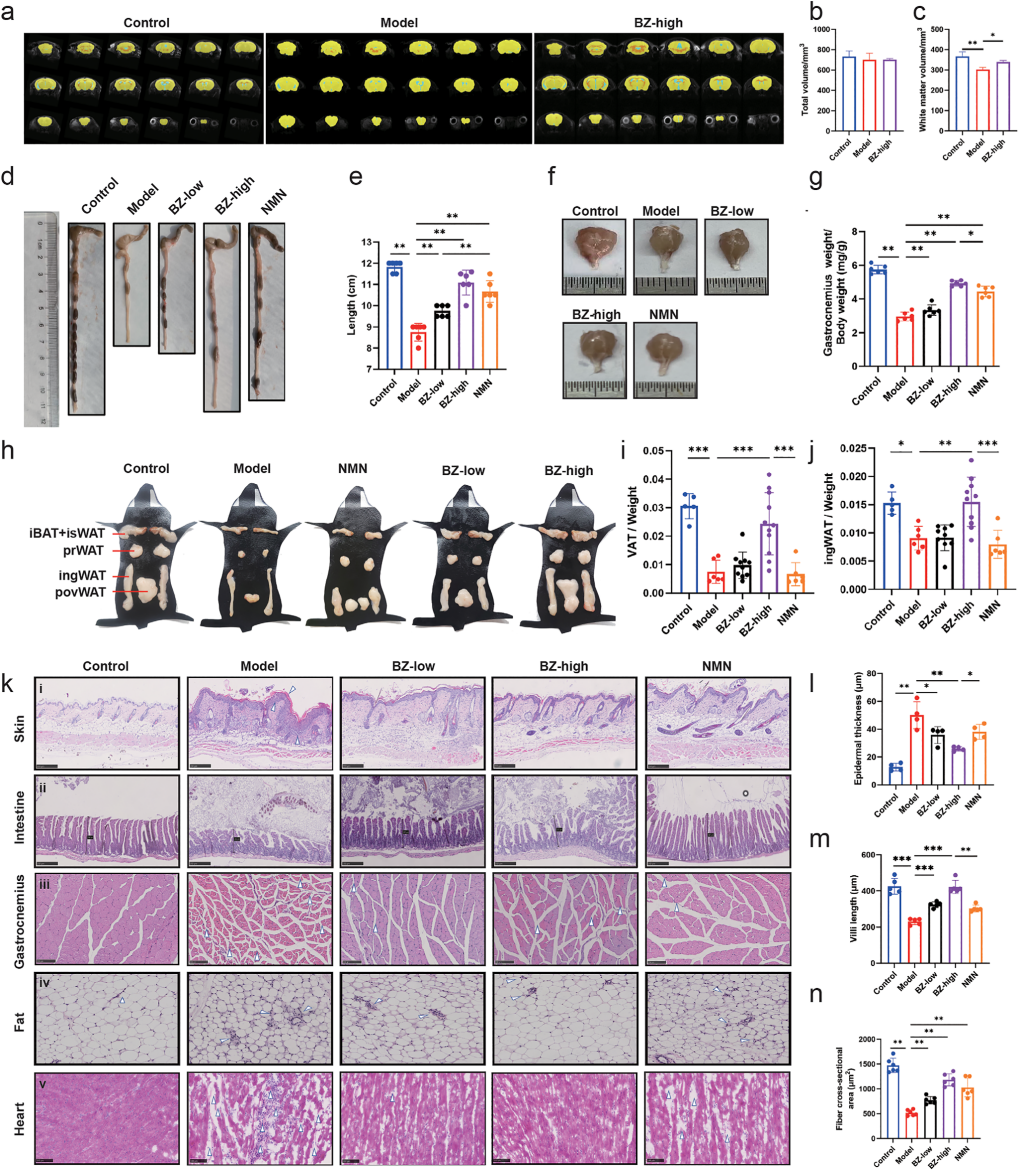
Chronic stress induces systemic histopathological alterations with variable therapeutic remediation. (a‐c) MRI analyses of brain morphology showing marked white matter atrophy in stressed mice, with partial recovery in BZBS. (n = 3). (d‐j) Gross anatomical quantification demonstrating stress‐induced (d, e) intestinal shortening (*n* = 6), (f, g) muscle mass loss (*n* = 6), and (h–j) adipose depletion, with differential drug efficacy i (*n* = 5‐11) j (*n* = 5‐10). (k i‐v) Representative histology: (i) Skin H&E staining showing epidermal thickening; (ii) Ileum pathology revealing villus atrophy; (iii) Muscle fiber disorganization quantified by cross‐sectional area irregularity. (iv) Adipose histopathology showing adipocyte atrophy. (v) Cardiac histopathology showing interstitial fibrosis. Arrows denote inflammatory cell infiltration. White arrows indicate inflammatory cell infiltration. (l‐n) Morphometric quantification of (l) epidermal thickness (*n* = 4), (m) villus height (*n* = 5), and (n) muscle fiber integrity (*n* = 6). Data presented as mean ± SD. Statistical significance determined by one‐way ANOVA with Tukey post hoc (multi‐group, ***p < 0.001, **p < 0.01, and *p < 0.05).

### 3.2 Chronic Stress Induces Myeloid‐Lymphoid Imbalance and Subpopulation Shifts at Single‐Cell Resolution

To study how long‐term chronic stress stimulation affects tissues at single‐cell resolution, we performed scRNA‐seq on key tissues from the central nervous system, digestive system, urinary system, respiratory system, and circulatory system, including the brain, small intestine, kidney, lung, and peripheral blood mononuclear cells (PBMCs) (Figure 3a). These organs exhibit a relatively higher diversity of cell types than others, and the microenvironments within the small intestine and lungs are even more complex. After quality control, we obtained 297,944 high‐quality cellular transcriptome datasets. Overall, we identified 51 main cell types across the samples (Figure 3b and Supplementary Table S7), with 20 of these cell types having tissue‐specificity, such as Hopx^+^ AT1 cells, and Sftpc^+^ AT2 cells in the lung (Figure 3c), Alpi^+^ intestinal epithelial cells in the small intestine (Figure 3d). The remaining 31 cell types were distributed across different tissues, such as Pecam^+^ endothelial cells (EC) in the brain, lung, and kidney, and Pdgfra^+^ fibroblasts (Fib) in the lung and kidney (Figure 3b–g). Ptprc^+^ immune cells were distributed across all five types of samples and were further divided into B cells, T cells, plasma cells, macrophages, monocytes, dendritic cells, etc (Figure 3b–g). In summary, we have generated a cross‐tissue single‐cell transcriptomic atlas of long‐term chronic stress for further analysis.

**FIGURE 3:**
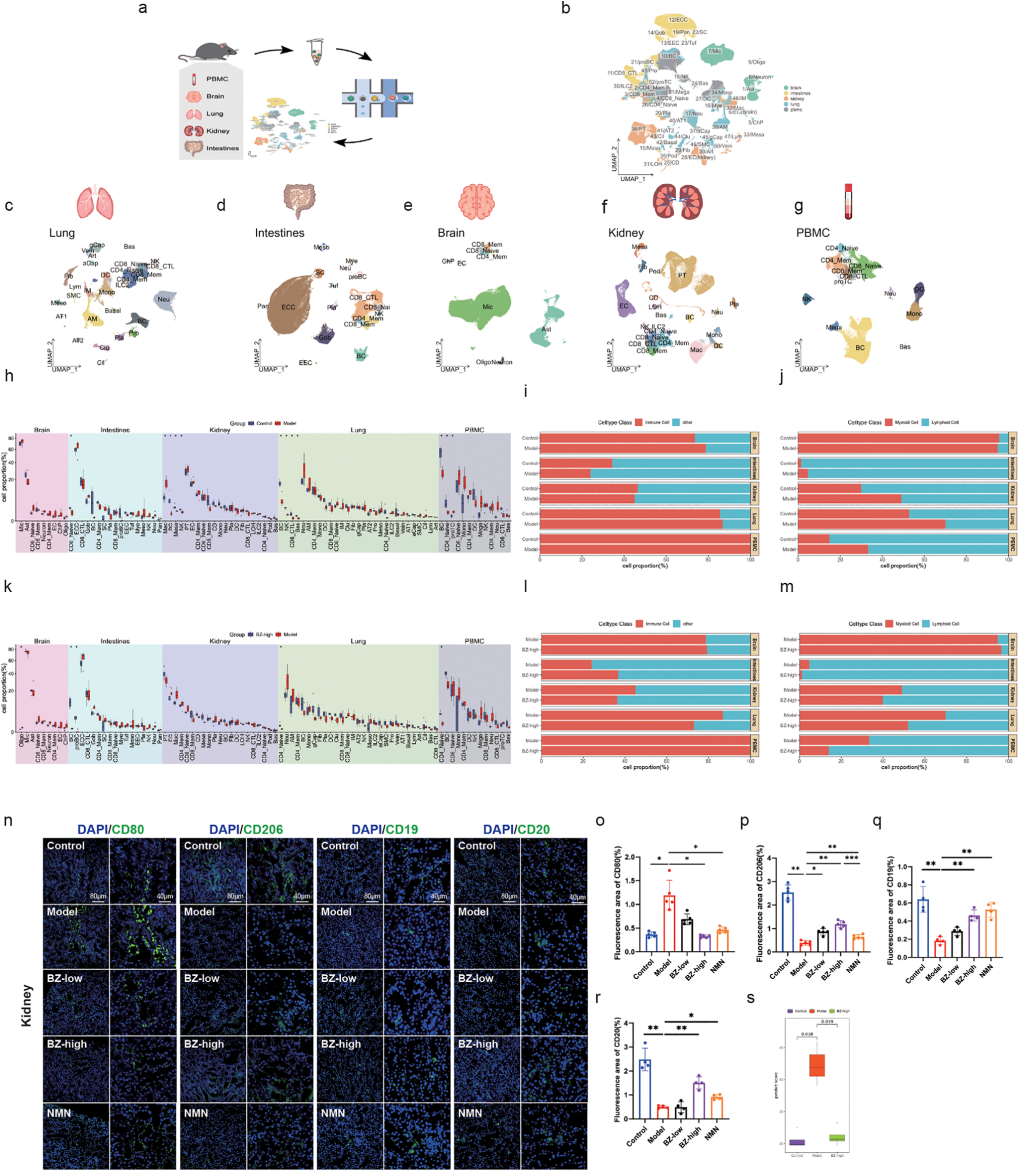
Single‐cell multi‐tissue transcriptomics reveal chronic stress‐induced myeloid‐immune imbalance. (a) Schematic of scRNA‐seq experimental design profiling brain, small intestine, kidney, lung, and PBMCs in chronic stress mice. (b‐g) Uniform Manifold Approximation and Projection (UMAP) visualization of single‐cell transcriptomes, annotating 51 cell types. aCap, aerocyte capillary; AM, alveolar macrophage; Art, artery; Ast, astrocyte; AT1, type I alveolar epithelial cell; AT2, type II alveolar epithelial cell; Bas, basophil; Basal; BC, B cell; CD, collecting duct; CD4_Mem, CD4^+^ memory T cell; CD4_Naive, CD4^+^ naive T cell; CD8_CTL, CD8^+^ cytotoxic T cell; CD8_Mem, CD8^+^ memory T cell; CD8_Naive, CD8^+^ naive T cell; ChP, choroid plexus cell; Cil, ciliated; Clu, club; DC, dendritic cell; EC, endothelial cell; ECC, enterocyte cell; EEC, en‐teroendocrine cell; Fib, fibroblast; gCap, general capillary; Gob, gobelt; ILC2, group 2 innate lymphoid cell; IM, Interstitial macrophage; LOH, loop of Henle; Lym, lymphatic; Mac, macrophage; Mega, megakaryocyte; Mesa, mesangial cell; Meso, mesothelial cell; Mic, microglia; Mono, monocyte; Mye, myeloid cell; Neu, neutrophil; Neuron; NK, natural killer cell; Oligo, oligodendrocyte; Pan, paneth; Pla, plasmocyte; Pod, podocyte; Pro, proliferating cell; proBC, pro‐B cell; proTC, pro‐T cell; PT, proximal tubule; SC, stem cell; SMC, smooth muscle cell; Tuf, tuft cell. (h) Box plot shows the distribution of different cell types across organs in the Control vs. Model group. (i, j) The proportions of immune cells vs. other cell types and myeloid cells vs lymphoid cells in Control vs Model group. (k) Box plot shows the distribution of different cell types across organs in the BZ‐high vs. Model group. (l, m) The proportions of immune cells vs. other cell types and myeloid cells vs. lymphoid cells in BZ‐high vs. Model group. (n‐r) Immunofluorescence staining of CD19^+^, CD20^+^, CD80^+^ and CD206^+^ immune cells in mouse kidneys (*n* = 5 for o, *n* = 5 for p, *n* = 4 for q, *n* = 4 for r). (s) siAge calculation of each group. *n* = 4‐5 for the biologically independent samples. Data presented as mean ± SD. Statistical significance determined by one‐way ANOVA with Tukey post hoc (multi‐group, ***p < 0.001, **p < 0.01, and *p < 0.05).

We observed that compared with somatic cells, the number of immune cells fluctuated significantly after long‐term chronic stress stimulation. Although the overall proportion of immune cells across the samples changed little, there was an imbalance in the proportion of myeloid/lymphoid immune cells, especially a significant reduction in the proportion of lymphoid immune cells, including B cells (BC), natural killer cells (NK), and T cells (TC) (Figure 3h–j). Decrease of BCs was obvious in most of the lung, kidney, and PBMCs (Figure 3h). Although the decrease in the proportion of BCs in the small intestine did not reach statistical significance, its average proportion dropped from 6.07% to 0.15%, a considerable reduction (Figure 3h), suggesting that BC may be one of the cell types most affected by long‐term chronic stress. The proportion of NK cells also significantly decreased in the kidney and lung (Figure 3h). The proportion of naive CD4^+^ and CD8^+^ T cells in PBMCs and small intestine significantly decreased (Figure 3h). Myeloid immune cells increased by 19.11%, 17.12%, and 18.32% in the kidney, lung, and PBMCs, respectively (Figure 3h,j). Especially, the proportion of macrophages significantly increased in the kidney (Figure 3h). The number of neutrophils in both the lungs and blood of the model group also exhibited a noticeable increase (Figure 3h). BZBS treatment restored the lymphocyte‐myeloid imbalance (Figure 3k–m). Immunofluorescence analysis confirmed that, in comparison to the Control group, the Model group displayed a marked elevation in the abundance of M1 macrophages (CD80^+^) across multiple tissues, whereas the number of M2 macrophages (CD206^+^) and B cells (CD19^+^ and CD20^+^) were significantly decreased (Figure 3n–r, Supplementary Figure S3). Treatment with BZBS and NMN resulted in a significant decrease in the number of M1 macrophages (CD80^+^) while concurrently enhancing the number of M2 macrophages (CD206^+^) and B cells (Figure 3n–r, Supplementary Figure S3). We employed the recently developed immune aging clock, siAge, to evaluate the degree of immune aging in mice [40]. Our findings indicate that the model group displayed considerable immunosenescence, while BZBS treatment significantly reduced this effect (Figure 3s).

We conducted subgroup analysis of B cells. Following the methodology established by Mathew et al. [39]. Tissue‐associated batch effects in this single‐cell dataset were corrected using Harmony software. The B cells segregated into eight distinct subclusters (Supplementary Figures 4a,b). Using AUCell, we quantified the activation levels of B cell (BC)‐related Gene Ontology (GO) gene sets (Supplementary Figures SS4c,d). Our analysis revealed that subcluster C0 principally aligned with mature marginal zone B cell differentiation, C1 corresponded to naive B cell differentiation, and C2 and C7 were linked to transitional states between immature and mature B cell differentiation (Supplementary Figure S4c). Subclusters C3‐C6 predominantly exhibited chemotaxis signatures and adhesion‐related phenotypes (Supplementary Figure S4c). Integration with Monocle 3 trajectory analysis and RNA velocity profiling demonstrated that C0 was at an early stage in the differentiation process (Supplementary Figures S4e,f). Compared to the control and treatment groups, stress‐challenged mice showed marked depletion of early differentiation‐stage C0 BCs and near‐complete loss of lung‐specific C6 BC subclusters, while the transitional C2 subcluster was greatly expanded (Supplementary Figures S4g–i). BZBS treatment reduced aberrant C2 subcluster while restoring C0 progenitor cells and intestinal tissue‐resident C4/C5 BC subgroups (Supplementary Figures S4g,h).

We also employed an analogous analytical framework to characterize macrophage and neutrophil subclusters within myeloid populations. Unsupervised clustering revealed 11 distinct macrophage (Mac) subgroups (Supplementary Figure S5a,b). Gene Ontology analysis identified specialized functional signatures: Mac C3/C4/C9 displayed strong associations with cytokine biosynthesis and granulocyte‐macrophage colony stimulating factors (GM‐CSF) signaling dynamics (production/response), whereas Mac C6 exhibited enrichment for chemotaxis regulation and migratory pathways (Supplementary Figure S5c). The expansion of C3/C4/C6 subclusters demonstrated enhanced proinflammatory polarization under stress stimulation, a pathological shift substantially mitigated by BZBS treatment (Supplementary Figure S5c–e). Notably, macrophages of the C3/C4 subtypes mostly existed in lung and kidney tissues (Supplementary Figure S5f), suggesting that they have a stronger inflammatory response under stress. On the other hand, neutrophil populations stratified into six transcriptionally distinct subclusters (Supplementary Figure S6a,b). Functional annotation revealed Neu C3 as the principal functional subcluster, harboring neutrophil differentiation and effector programs (Supplementary Figure S6c). Integrative computational modeling combining pseudotemporal ordering with transcriptional dynamics demonstrated stress‐induced accumulation of Neu C3 during primitive differentiation phases (Supplementary Figure S6d–g). Similarly, pharmacological intervention with BZBS effectively normalized this pathological expansion (Supplementary Figure S6f,g). Notably, the lungs housed the largest proportion of neutrophil subtypes (Supplementary Figure S6h). Previous studies indicate that the lung retains sequestered leukocytes and serves as a reservoir for hematopoietic progenitors, which play a critical role in immune and inflammatory regulation [45, 46, 47]. By incorporating immune cell analysis, we revealed the changes in immune defense efficiency and inflammatory response resource allocation within the organ's immune microenvironment under stress, suggesting a potentially significant role for the lungs (Supplementary Figure S6i).

### 3.3 Tissue‐Specific Transcriptional Programs Drive Chronic Stress‐Accelerated Inflammaging

Analysis of intercellular communication revealed immune cells as dominant mediators of dysregulated cell‐cell signaling networks (Figure 4a). Notably, key dynamic cell types—intestinal stem cells and lung AT2/ciliated cells—showed strong myeloid regulation (neutrophils, monocytes, and macrophages), and lung venous and smooth muscle cells were similarly regulated (Figure 4a and Supplementary Figure S7a,b). More surprisingly, BZBS almost completely reversed the abnormal immune cell‐somatic cell interaction patterns caused by stress (Figure 4a and Supplementary Figure S7a,b). We further evaluated cytokines and inflammatory status across organs. Quantitative PCR validation confirmed substantial upregulation of inflammatory and senescence markers—including *NF‐κB*, *TNF‐α*, *IL‐6*, *P16^INK4a^*, *P21*, and *P53*—across multiple tissues in the Model group. High‐dose BZBS treatment effectively suppressed these transcriptional increases, whereas BZ‐low and NMN groups exhibited comparatively muted improvements (Supplementary Figure S8a–n). Immunofluorescence staining of brain tissues further corroborated these findings, demonstrating elevated Iba‐1 expression indicative of pro‐inflammatory microglial activation in the Model group (Supplementary Figure S8o,p). Contrasting with confinement of cell number alterations predominantly to immune populations, AUCell analysis using predefined inflammatory response gene signatures revealed that UCMS significantly elevated inflammation in all samples and most cell subtypes, especially AT2 and fibroblasts in the lung, PT and fibroblasts in the kidney, neurons, microglia, CHP cells in the brain, neutrophils, stem cells, and other important functional somatic cells in the intestine (Figure 4b,c). BZBS reduced the level of inflammation in PBMCs at the overall level and reversed the level of inflammation in AT2, fibroblasts, and SMCs in the lung at the cellular level (Figure 4d,e).

**FIGURE 4:**
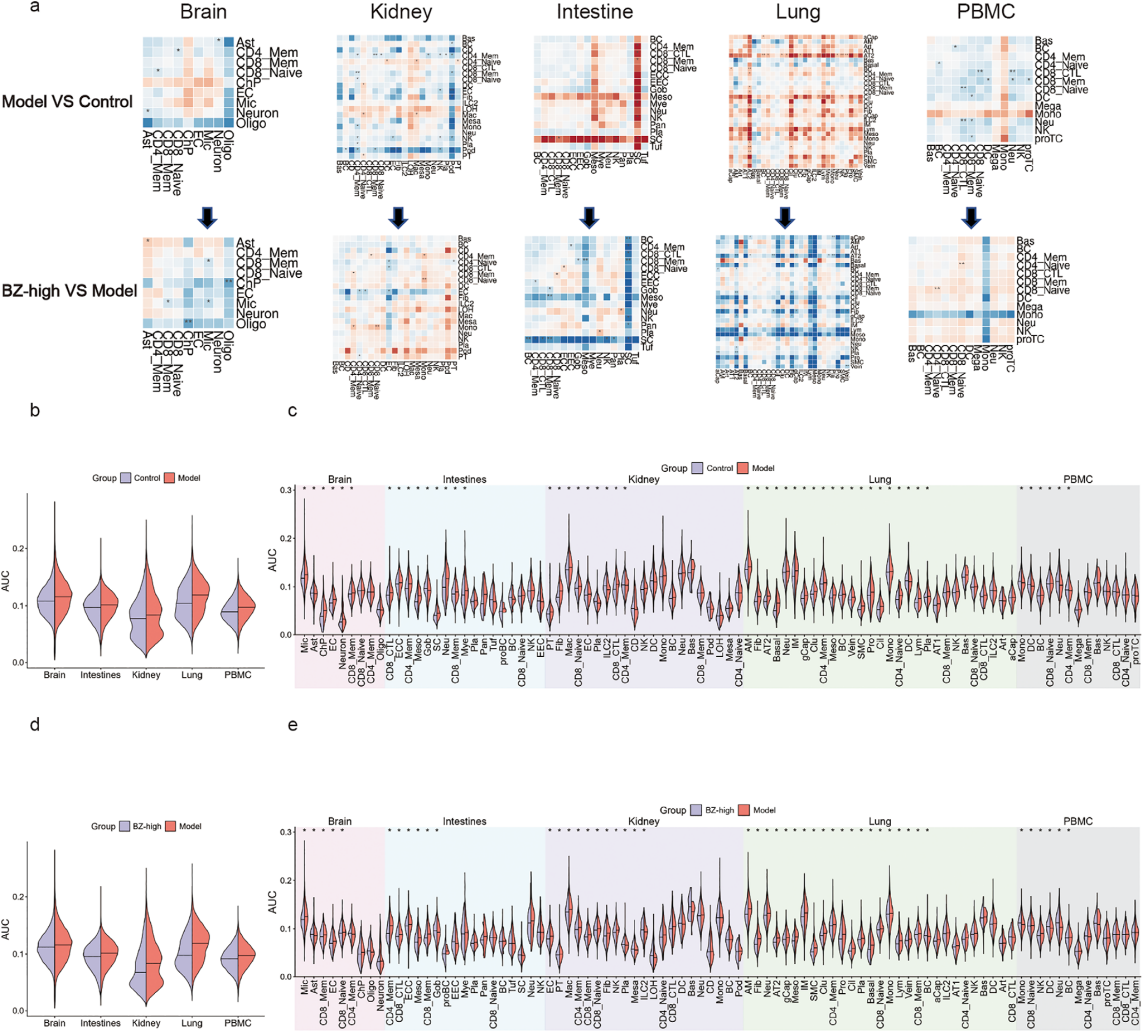
Immune dysregulation propagates multi‐tissue inflammatory aging. (a) Cell‐cell communication: Stress amplified immune‐to‐somatic signaling. BZBS restored baseline interaction patterns. (b) AUCell quantification of inflammatory‐DEGs in tissues of each group. (c) AUCell quantification of inflammatory‐DEGs in in each type of cell. (d, e) Tissue‐ and cell‐specific inflammatory scores quantified by AUCell following BZBS treatment.

To compare the effects of stress across tissues, we characterized stress‐related differentially expressed genes (S‐DEGs) in each tissue and observed their uneven distribution. In absolute terms, the lung and kidney exhibited the highest number of S‐DEG (Figure 5a,b). After normalization relative to cell type abundance, the lung retained the highest proportion of S‐DEG, followed by peripheral blood mononuclear cells (PBMCs) (Figure 5c). AUCell analysis confirmed significantly elevated stress levels in PBMCs (Figure 5d). Functional analyses revealed that upregulated S‐DEG primarily involved innate immune responses, leukocyte activation, and apoptotic regulation, with prominent enrichment in the lung, kidney, and PBMC populations (Figure 5e,f). Notably, PBMCs uniquely exhibited enrichment in protein folding and maturation pathways, while the lung displayed enhanced cellular response to stimuli. Conversely, downregulated S‐DEGs were associated with adaptive immunity, antigen processing/presentation, and cell adhesion mechanisms (Figure 5g,h). These results corroborated our previous findings of innate immune activation concurrent with adaptive immune suppression, demonstrating chronic stress‐induced immune reprogramming. It is worth noting that there are 73 common genes among the five organs, such as the upregulated Apoe and Mt1 genes (Supplementary Figure S9a). The senescence marker gene Cdkn1a (P21) was also upregulated in multiple cell types (Supplementary Figure S9b). Some genes were downregulated, mainly including B2m, Ifi27l2a, and the H2 family (Supplementary Figure S9b). BZBS treatment effectively reduced stress levels, especially in the lung and the kidney (Supplementary Figure S10a).

**FIGURE 5:**
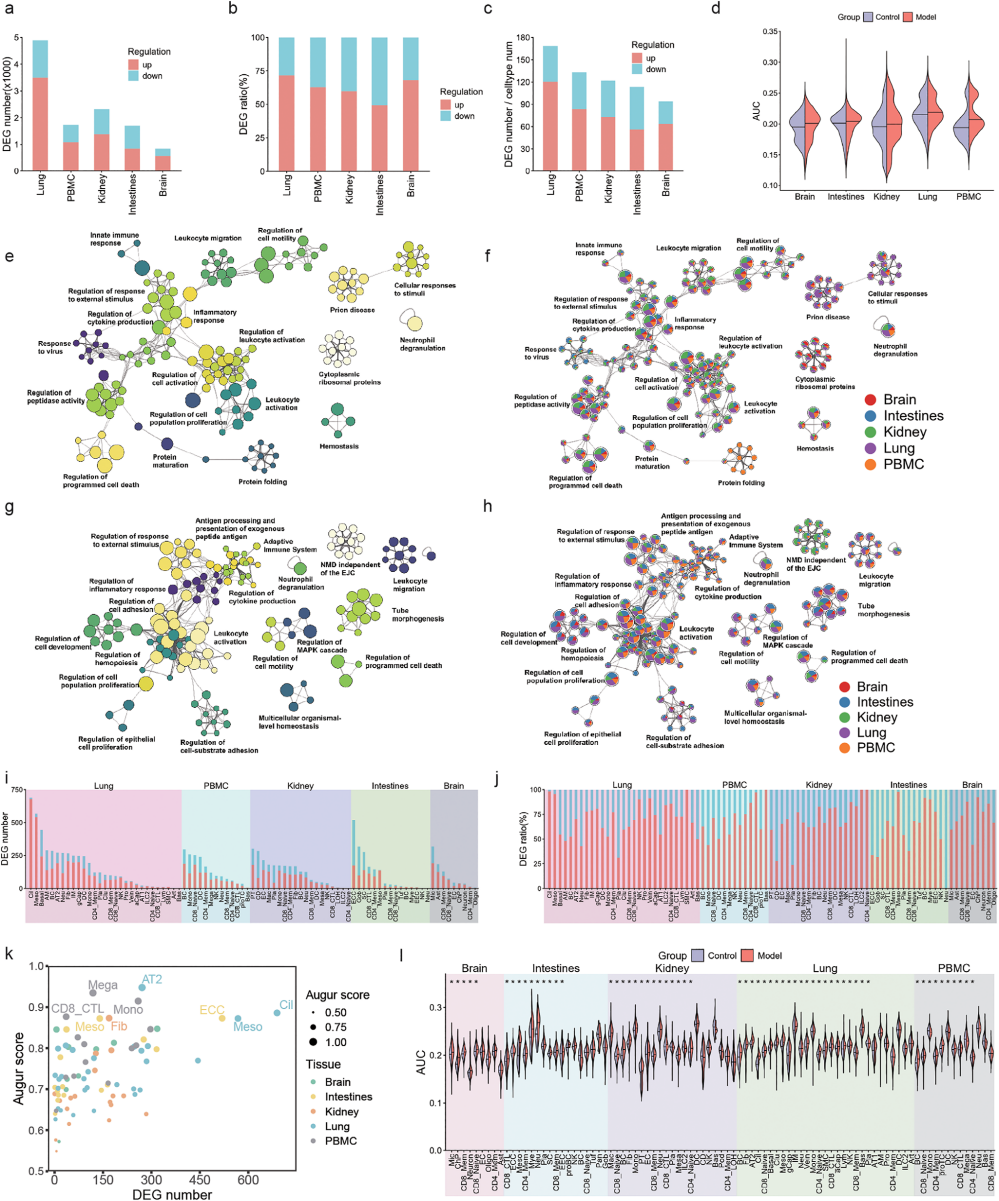
Tissue‐specific transcriptional rewiring under chronic stress. (a–c) Cross‐tissue stress‐induced differential gene expression. (d) AUCell quantification of stress scores. (e–h) Functional enrichment: (e, f) Upregulated pathways in different tissues; (g, h) Downregulated pathways in different tissues. (i–l) Stress‐induced differential gene expression of multi‐cell types cross‐tissue: (i, j) Expression proportion of stress‐DEGs in each cell type of tissues; (k) Augur score of stress‐DEGs in each cell type of each tissue; (l) AUCell quantification of stress‐DEGs in each cell type of each tissue.

Correlation analysis showed that the stress level of most cells was positively correlated with the inflammation level (Supplementary Figure S10b). Non‐immune cell types (excluding PBMCs) exhibited the highest S‐DEG quantities and stress magnitudes, particularly pulmonary ciliated cells (Cil), renal proximal tubular (PT) cells, and small intestinal enterochromaffin (ECC) cells (Figure 5i,j). This phenomenon was further validated through Augur‐based analysis of global transcriptional shifts and AUCell analysis of stress level, identifying Cil, mesothelial (Meso), and alveolar type II (AT2) cells in the lung and ECCs in the intestine as key responders to chronic stress (Figure 5k,l). BZBS treatment effectively reversed the stress levels in these cells (Supplementary Figure S10c,d).

### 3.4 Multidimensional Characterization of Tissue‐ and Cell‐Aging Heterogeneity

Immunosenescence and inflammation constitute a key driver of aging. Single‐cell transcriptomic and quantitative PCR analyses revealed multi‐organ upregulation of senescence‐associated genes such as *P21* and *P16^INK4a^*. To systematically evaluate the association between transcriptional changes and aging pathology, we intersected stress‐induced differentially expressed genes (DEGs) with curated aging‐related gene signatures [48, 49], defining a subset of “stress‐pro‐aging‐DEGs (SA‐DEGs)” (Figure 6a). Across tissues, pulmonary and peripheral blood mononuclear cell (PBMC) populations exhibited the highest counts of SA‐DEGs, while cerebral tissues—despite fewer total stress DEGs—demonstrated the strongest aging signature overlap (86.7% aging‐related gene proportion) (Figure 6b–d). AUCell quantification identified kidneys and PBMCs as displaying the most pronounced elevation in aging scores (Figure 6e). In terms of the number and proportion of stress aging genes, in addition to PBMs, the lungs also have a large number of SA‐DEGs cells, such as ciliated cells, mesothelial cells, basal cells, AT2, and fibroblasts (Figure 6f,g). AUCell analysis also showed that important somatic cells, stem cells, B cells, macrophages, and CD8^+^ cytotoxic T lymphocytes (CTL) in the lungs showed obvious aging upgrades (Figure 6h). Notably, aging patterns exhibited tissue‐specific regulation: pulmonary and intestinal CD8^+^ cytotoxic T lymphocytes (CTLs) displayed marked senescence escalation, whereas CD4^+^ memory T cells showed compartmentalized changes—reduced senescence in PBMCs/kidneys but elevated levels in the intestine (Figure 6h). B lymphocytes demonstrated the most robust pan‐tissue senescence amplification, with significant increases observed across pulmonary, renal, and PBMC compartments (Figure 6h). We also defined genes whose trends in BZ‐high versus Model were opposite to SA‐DEGs as BZ‐rev‐aging‐DEGs (BRA‐DEGs). The lung had the most absolute BRA‐DEGs, while the PBMCs produced the most BRA‐DEGs after cell type correction (Supplementary Figure S11a–c). The intestine had the highest total and BZBS‐specific DEG number, and the brain had the highest proportion of BRA‐DEGs (Supplementary Figure S11a–c). BZBS reduced the aging scores in the PBMCs (Supplementary Figure S11d). The action of BZBS has clear heterogeneity in different organs and cell types, with some cell types showing high gene expression changes, such as BCs, Mono, and astrocytes in the brain (Supplementary Figure S11e,f). BZBS specifically reduced the aging scores in multiple cell types in the lungs, kidneys, and intestines (Supplementary Figure S11g).

**FIGURE 6:**
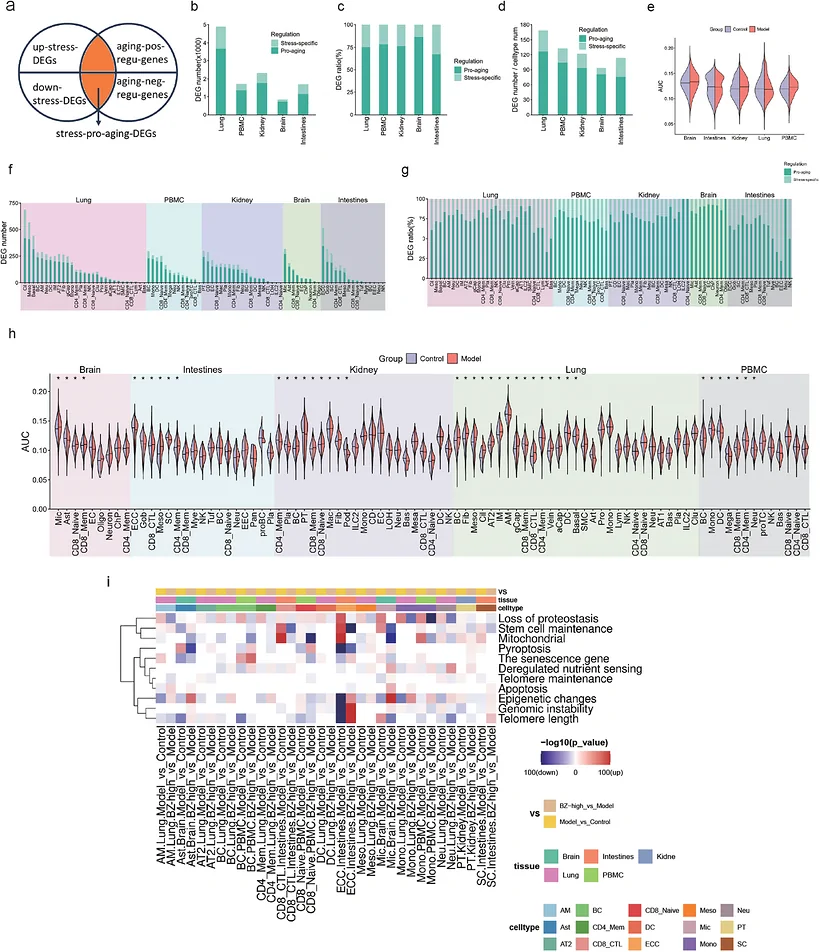
Multidimensional mapping of stress‐driven aging heterogeneity. (a–d) Proportion of stress‐induced pro‐aging differentially expressed genes (DEGs) in each tissue. (e) AUCell aging scores across tissues. (f, g) Distribution of stress‐induced pro‐aging DEGs across different cell types. (h) AUCell aging scores across different cell types. (i) Scoring of aging hallmarks in various tissue cell types.

To systematically identify stress‐modulated aging hallmarks, we curated gene sets associated with aging characteristics—including genomic instability, telomere shortening, epigenetic alterations, protein homeostasis imbalance, autophagy dysfunction, mitochondrial dysfunction, nutrient sensing defects, cellular senescence, and stem cell exhaustion—along with cell death pathways (apoptosis and pyroptosis) from KEGG, MGI, and GO databases for enrichment scoring. Our findings indicate that the majority of cellular aging hallmarks are predominantly associated with mitochondrial dysregulation, epigenetic alterations, and senescence‐related genes, while PT cells in the kidney, SC and ECC cells in the intestine, and most immune cells had almost all senescence characteristics (Figure 6i). Consistent with the mitochondrial scoring results, we detected high levels of lipid and protein peroxides in various organs of mice in the model group (Supplementary Figure S12a,b). Quantitative analysis also revealed significant glutathione depletion and impaired SOD activity in tissues of stressed mice, demonstrating systemic redox imbalance (Supplementary Figure S12c,d). These biochemical perturbations were substantially attenuated in BZBS‐treated animals. Moreover, Emerging consensus evidence demonstrates that activated innate immune cells significantly contribute to oxidative damage through multiple pathways [50, 51, 52]. In line with our analysis of oxidative stress characteristics in pulmonary immune cells, immune fluorescence staining of lungs from aging‐model mice revealed that lung macrophages exhibited significantly elevated mtROS levels in the model group, while BZBS treatment effectively reduced mtROS in these immune cells (Supplementary Figure S13).

At the epigenetic level, RRBS profiling of liver tissues revealed genome‐wide methylation increases in model animals, showing distinct enrichment patterns in genic elements (particularly introns), 3'UTR regions, and GC‐rich genomic compartments including CpG islands (Supplementary Figure S14a) along with accelerated epigenetic aging (Supplementary Figure S14b)—phenotypes that were significantly reversed by BZBS treatment. Together these data established BZBS's multimodal protective effects against aging‐associated pathophysiology.

### 3.5 BZBS Combats Mitochondrial Stress Through AMPK‐Activating Compounds

A total of 23 prototypes were detected in rat plasma, and 5 prototypes were detected in rat brain after the oral administration of BZBS (Figure 7a, Supplementary Figure S15, and Table 8). These chemicals are predominantly derived from *Schisandra chinensis*, *Cnidium monnieri*, *Cuscuta australis*, *Lycium chinense Miller*, *Rubus chingii*, *Rosa laevigata*, and *Epimedium* species, representing potential therapeutic mediators for BZBS's pharmacological activity. Building upon prior characterization of aging‐related tissue and cellular alterations, along with established bioactivity profiles of BZBS, we selected rat brain astrocyte cells CTX and mouse mononuclear macrophage leukemia cells Raw264.7 for mitochondrial injury modeling using hydrogen peroxide‐ or CCCP‐mediated respiratory inhibition protocols. Screening of brain‐ and blood‐derived compounds identified that Osthole, Schisandrin, and Schisandrin B exhibited dose‐dependent mitigation of astrocyte mitochondrial dysfunction. These compounds not only suppressed mitochondrial reactive oxygen species (mtROS) overproduction but also restored mitochondrial membrane potential (Figure 7b–f and Supplementary Figure S16). Notably, Osthole further enhanced mitochondrial respiratory capacity (Figure 7g‐k). And co‐treatment with 10 µM of Osthole and Schisandrin B synergistically inhibited cellular mtROS (Figure 7l). In parallel experiments in the Raw264.7 mitochondrial damage model, Osthole, Schisantherin A, and Schisandrin B exhibited mtROS‐quenching properties (Figure 7m,n). In addition, Schisantherin A and Schisandrin B also attenuated radiation‐induced genomic instability in immune cells (Figure 7o,p). Notably, synergistic interactions among these compounds were observed in irradiation‐induced senescence models. Combined treatment (Osthole + Schisantherin A + Schisandrin B) outperformed individual compounds in attenuating DNA damage (Supplementary Figure S17a,b) and cellular senescence (Supplementary Figure S17c,d). These findings collectively establish BZBS as a multicomponent therapeutic repository containing multiple bioactive agents capable of reversing hallmark aging phenotypes. For mechanistic benchmarking, we compared BZBS with two clinically relevant anti‐aging compounds—NMN (NAD^+^ booster) and ABT‐263 (senolytic). In our irradiation‐induced senescence Raw264.7 model, both BZBS and NMN effectively reduced mitochondrial ROS (mtROS) and attenuated DNA damage (γH2AX) (Supplementary Figure S18). Although ABT‐263 also reduced senescence‐associated markers, its effects were primarily mediated through selective elimination of senescent cells, as evidenced by significantly fewer surviving cells in the ABT‐263 group compared to BZBS and NMN treatment (Supplementary Figure S18). These results provide evidence for evaluating BZBS's efficacy while highlighting mechanistic differences among anti‐aging compounds.

**FIGURE 7:**
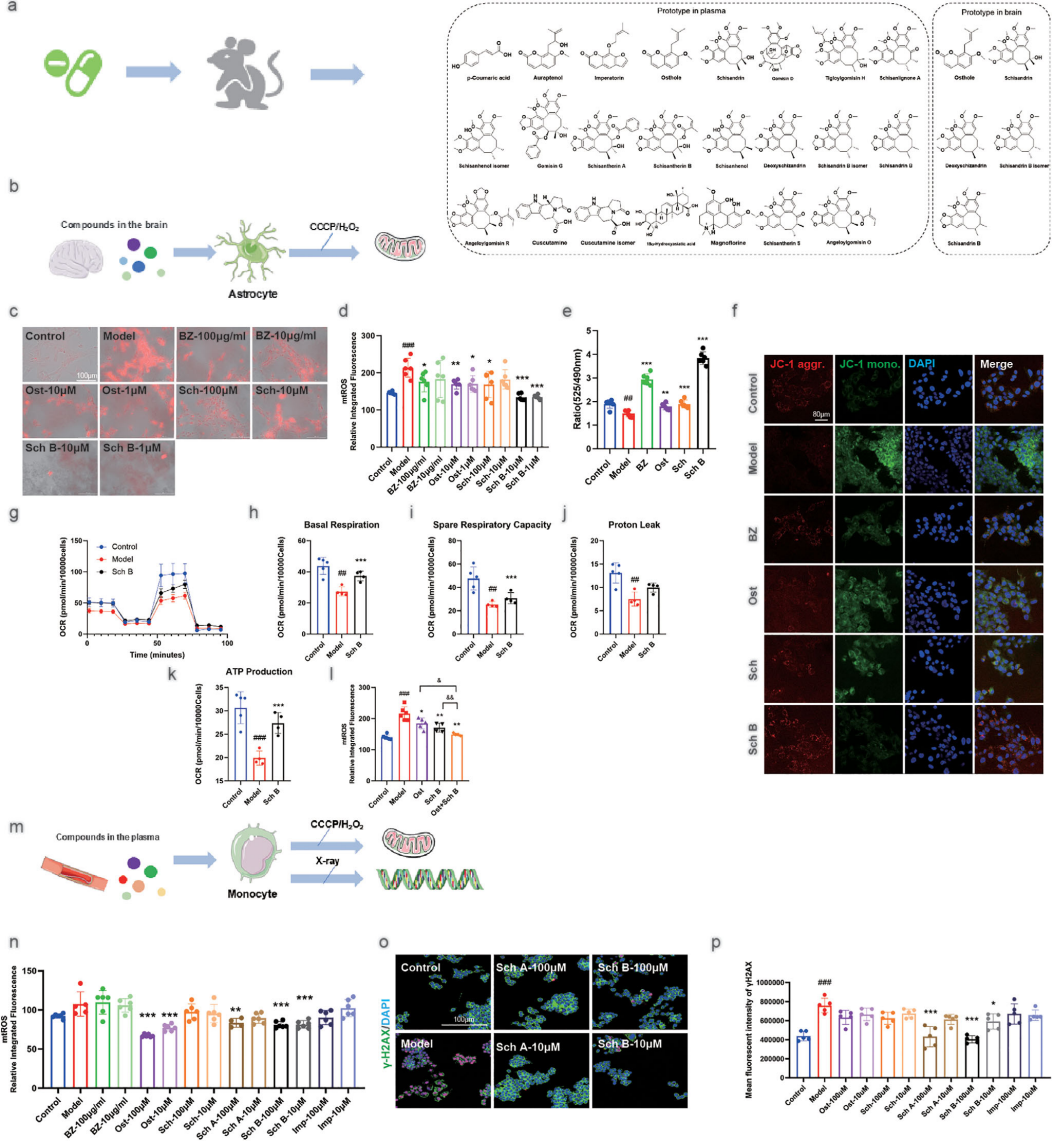
Active compounds in BZBS ameliorate mitochondrial dysfunction and genome instability in astrocytes and immune cells. (a) Pharmacokinetic profile of BZBS in rats. (b) Schematic illustrating the induction of oxidative stress and mitochondrial damage in astrocytes. (c, d) Effects of brain‐penetrating drug components of BZBS on the reduction of mitochondrial ROS in astrocytes (*n* = 5‐6). (e, f) Effects of brain‐penetrating drug components of BZBS on the preservation of astrocyte mitochondrial membrane potential (*n* = 6). (g‐k) Effects of Osthole on enhancing mitochondrial respiration in astrocytes (*n* = 3 for g, *n* = 4‐5 for h, *n* = 4‐5 for i, *n* = 4‐5 for j, *n* = 4‐5 for k). (l) Synergistic effect of Osthole and Schisandrin B in reducing cellular mtROS (n = 4‐5). (m) Schematic of the experimental model for inducing mitochondrial damage and genomic instability in monocytes. (n) Effects of systemically circulating drug compounds of BZBS on the reduction of mitochondrial ROS in monocytes (*n* = 4‐6). (o, p) Protective effects of systemically circulating drug compounds of BZBS on monocyte genomic integrity following X‐ray irradiation (*n* = 5). Data are expressed as mean ± SEM. Statistical significance was assessed by one‐way ANOVA with Tukey's post hoc test. Asterisks (*) indicate treatment vs. vehicle control (***p < 0.001, **p < 0.01, and *p < 0.05). Hashtags (#) indicate model group vs. untreated control (###p < 0.001, ##p < 0.01, and #p < 0.05). Ampersands (&) indicate comparison between treatment groups (&&&p < 0.001, &&p < 0.01, and &p < 0.05).

To elucidate the molecular targets underlying the mitochondrial regulatory effects of these compounds—initially selected based on their superior efficacy in mitigating mitochondrial ROS, restoring membrane potential (Osthole, Schisandrin, Schisantherin A, and Schisandrin B), and enhancing respiratory function (Osthole) across CTX astrocyte and Raw264.7 macrophage models—comprehensive compound‐target networks were established for Osthole, Schisandrin, Schisantherin A, and Schisandrin B, followed by systematic curation of oxidative stress and epigenetic regulation‐related targets (Supplementary Figure S19a). Computational intersection identified 414 shared targets (Supplementary Figure S19b), with protein‐protein interaction and biological process enrichment analysis revealing a predominant involvement of antioxidative stress genes, including *PPARGC1A* (coding PGC‐1α), *PRKAA2* (coding AMPK), and *Sirtuin 1* (Supplementary Figure S19c–e). We subsequently validated this computational prediction through pharmacological interrogation using two complementary models: astrocytes and immune cells subjected to mitochondrial stress challenges. Co‐treatment with 20 µM AMPK inhibitors compound C effectively antagonized the mitochondrial protective effects of Schisantherin A in CTX cells (Figure 8a,b) and Raw264.7 cells (Figure 8c). To further validate the essential role of AMPK in mediating BZBS's effects, we performed genetic knockdown experiments in Raw264.7 cells and CTXs (Supplementary Figure S20, S23). Successful AMPKα1 or AMPKα2 knockdown significantly attenuated BZBS‐derived compounds' ability to mitigate mitochondrial dysfunction, as evidenced by persistent mtROS accumulation and failure to restore DNA Integrity (Supplementary Figure S20d–g). Molecular docking simulations suggested favorable binding affinities between all evaluated compounds and AMPK catalytic domains (Supplementary Figure S21a–d). Osthole and Schisandrin B exhibit potential binding affinity for SIRT1 (Supplementary Figure S21e,f). Additionally, Schisantherin A and Schisandrin B show potential binding affinity toward SIRT3 (Supplementary Figure S21g,h) while Schisandrin B may also bind to SIRT6 (Supplementary Figure S21i). These Sirtuins (SIRT1/3/6) serve as key effectors in the AMPK‐mitochondrial signaling axis. Subsequent BLI assays corroborated these findings: all compounds displayed robust binding to AMPKα1 (*K*_d_ = 10.305 µM to 228.801 µM, Figure 8d), and AMPKα2 (*K*_d_ = 2.09 µM to 89.8 µM, Figure 8e), with Osthole and Schisandrin B further confirming interactions with SIRT1 (*K*_d_ = 241 µM and 10.9 µM, Figure 8f). Notably, Schisantherin A and Schisandrin B exhibited additional binding to SIRT3 (*K*_d_ = 164 µM and 40.4 µM, Figure 8f), whereas Schisandrin B uniquely demonstrated detectable affinity for SIRT6 (*K*_d_ = 152.1 µM, Figure 8f). To validate target engagement under physiological conditions, we performed cellular thermal shift assays. Treatment with 20 µM of BZBS's active components significantly elevated the thermal stability of endogenous AMPKα1/α2, SIRT1, SIRT3, and SIRT6, confirming direct intracellular binding (Supplementary Figures S22, S24). Crucially, cellular treatment with these compounds consistently enhanced AMPK enzymatic activity, an effect that was abolished by AMPK inhibition (Figure 8g). Furthermore, Osthole and Schisandrin B promoted nuclear translocation of PGC‐1α and SIRT1 in CTX cells, an effect reversed by AMPK inhibition (Figure 8h,i). To confirm these interactions at the protein level, we performed Western blot analyses in lung tissues from BZBS‐treated mice. These experiments demonstrated that BZBS significantly increased phosphorylation of AMPK (p‐AMPKα/total AMPK), consistent with AMPK activation, and upregulated protein expression of SIRT1, SIRT3, and PGC‐1α (Figure 8j–m and Supplementary Figure S23), directly aligning with our cellular findings. Collectively, these multimodal analyses identify Schisandrin derivatives as the primary bioactive constituents through which BZBS ameliorates stress‐induced mitochondrial dysfunction, primarily via activation of the AMPK‐SIRT‐PGC‐1α signaling axis.

**FIGURE 8:**
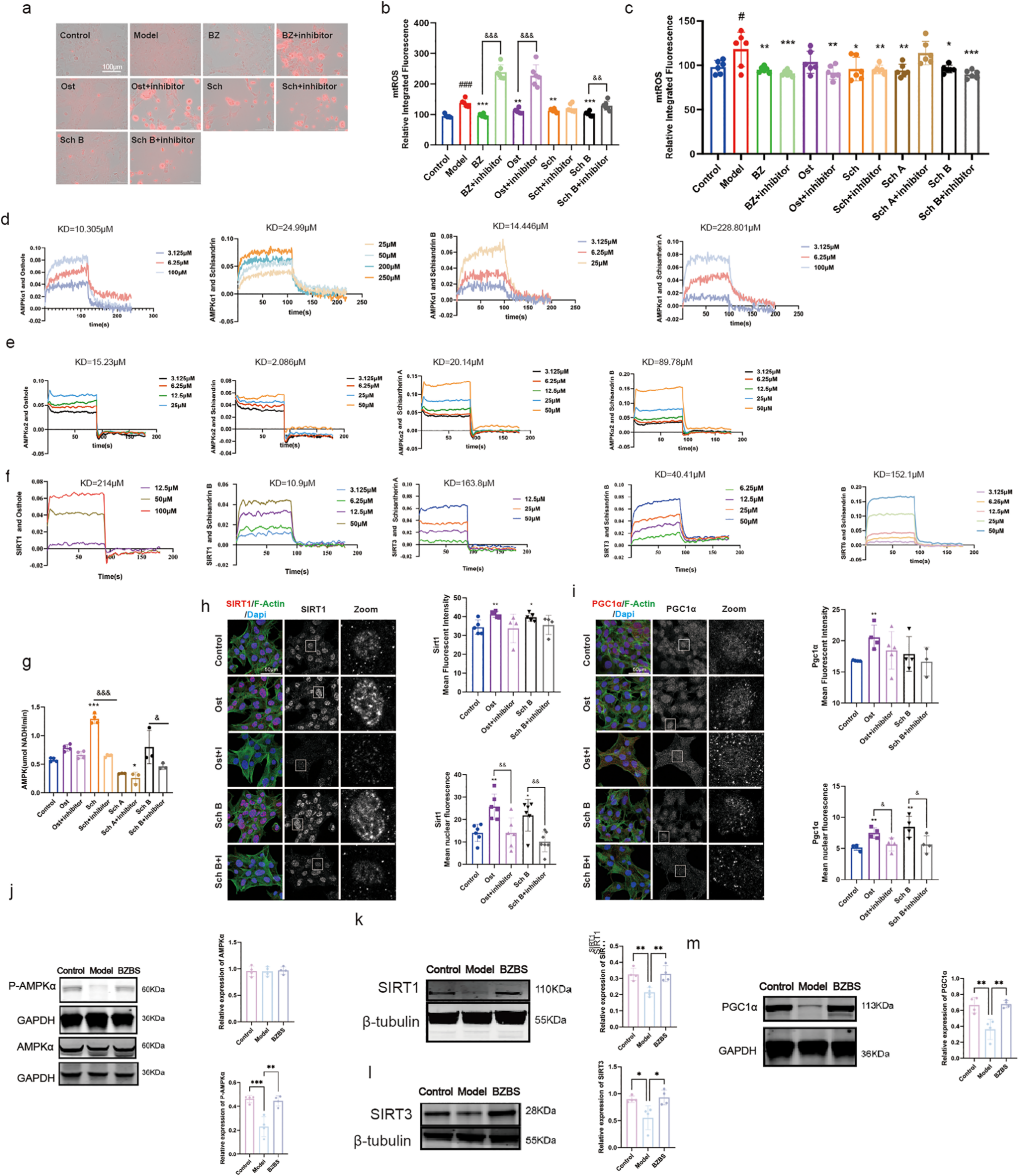
Active compounds from *Cnidium monnieri* and *Schisandra chinensis* directly target AMPK to improve mitochondrial function. (a–c) Pharmacological inhibition of AMPK with compound C reversed the protective effects of BZBS ingredients on mitochondrial function in (a, b) CTX cells (*n* = 4‐6) and (c) RAW264.7 cells (n = 6). (d–f) BLI confirmed direct binding of BZBS components to AMPKα1 (d) and AMPKα2 (e), with Osthole and Schisandrin B also interacting with SIRT1, Schisantherin A and Schisandrin B with SIRT3, and Schisandrin B uniquely with SIRT6 (f) (*n* = 3). (g) AMPK activity measurement demonstrated that BZBS ingredients promoted AMPK activity (*n* = 3‐4). (h, i) BZBS ingredients facilitated nuclear translocation of PGC‐1α and SIRT1, effects that were abolished by AMPK inhibition (*n* = 5‐7 for h, *n* = 3‐5 for i). (j‐m) Western blot analysis of lung tissues from BZBS‐treated mice confirmed in vivo activation of the AMPK‐PGC‐1α‐SIRT pathway (*n* = 4). Data are presented as mean ± SEM. Statistical significance was determined by one‐way ANOVA followed by Tukey's post hoc test. Asterisks (*) indicate treatment vs. vehicle control (). Hashtags (#) indicate model group vs. untreated control (###p < 0.001, ##p < 0.01, and #p < 0.05). Ampersands (&) indicate comparison between treatment groups (&&&p < 0.001, &&p < 0.01, and &p < 0.05).

## 4 Discussion

Traditional aging studies predominantly rely on chronological aging models under normal feeding conditions. While these models capture fundamental aging trajectories, they are constrained by the time‐consuming nature of longitudinal studies and the relative simplification of mechanistic pathways, compared to the real social environment. In contrast, the chronic stress model employs a pathological “time compression” strategy to recapitulate the complex aging network characterized by multisystem interactions within a defined experimental timeframe. This approach not only elucidates organ‐specific vulnerability profiles but also unearths critical aging drivers that are often obscured in conventional aging paradigms. The accelerated phenotype generation enables in‐depth investigation of clinically relevant mechanisms intersecting psychosocial stressors and biological aging. In UCMS mice, we established causal links between chronic stress exposure and accelerated phenotypic aging, quantified through decline trajectories (functional deterioration, elevated FI scores) and compressed survival curves (Figure 1b–o). Multi‐organ histopathogical mapping revealed systemic erosion of tissue integrity (Figure 2 and Supplementary Figure S2). Notably, we observed widespread inflammatory cells in multiple tissues of UCMS mice, with upregulated expression of related inflammatory cytokines and increased senescence‐related markers, suggesting that systemic low‐grade inflammation and accumulation of senescent cells are prominent features of accelerated aging in long‐term UCMS, closely related to “inflammaging” [53, 54].

Characteristics of immune system aging include decreased lymphocyte production, decreased adaptive immunity, increased inflammation, etc. [55, 56]. Previous studies conducted by Yousefzadeh et al. (2021) suggest that accelerated aging of immune cells following gene editing poses the greatest health risk and exacerbates damage to solid organs [57]. However, under physiological conditions, the precise trigger of accelerated immune aging remains elusive. We found that UCMS caused extensive immune system aging, not only evident in elevation of myeloid immune cells and decline in lymphoid immune cells but also upregulation of a wide range of stress, inflammatory, and aging genes. Marked depletion of naive/intermediate B cells indicative of BC exhaustion elevated infection risks, which mirrors social stress‐induced immunosuppression (Supplementary Figure S4). On the other hand, myeloid populations broadly affect tissue‐resident stem/somatic cells—lung AT2/ciliated, kidney PT, and intestinal stem cells—emerging as central drivers (Figure 4a and Supplementary Figure S7). Collectively these data reveal disrupted immune homeostasis as central to stress‐accelerated aging. Notably, Heidt et al. previously demonstrated UCMS‐induced myeloid skewing of hematopoietic stem cells in bone marrow [8]. While this suggests marrow hematopoietic reprogramming, the precise origin of systemic immune dysregulation (stem cell‐intrinsic alterations vs. secondary tissue microenvironment effects) requires further investigation. Critically, as previous studies reported, this immune dysfunction directly fuels oxidative tissue damage through two key mechanisms: Infiltrating macrophages elevate mitochondrial ROS via NOX_2_‐dependent pathways, driving protein carbonylation and DNA oxidation [50, 52], and neutrophil‐derived MPO generates hypochlorous acid that amplifies lipid peroxidation [58, 59]. Our renal data show an mtROS increase in UCMS macrophages (Supplementary Figure S13), while BZBS reverses this cascade, highlighting its therapeutic potential of breaking the immune‐oxidative cycle in stress‐accelerated aging. Future studies employing cell‐specific knockouts will further delineate these mechanisms.

Regarding different responses of tissues to stress, while the blood‐brain barrier restricts immune penetration (T/B cell paucity), brain transcriptional shift is predominantly mapped to aging‐related pathways (Figure 6b–d), revealing the UCMS‐induced neural aging axis through immune‐prioritized mechanisms. On the other hand, the lung, as an open organ directly exposed to the external environment, requires complex immune defense mechanisms to protect the body from pathogens. Among organs characterized by complex cellular composition, the lung exhibits most UCMS‐induced vulnerability through profound immune remodeling and stem/somatic cell alterations (Figure 6). Our data, along with prior studies, indicate that the lung serves as a critical regulatory site for immune cell dynamics [45, 46, 47]. These characteristics may render the lung one of the organs most susceptible to immune cells, potentially functioning as a hub for senescence propagation. Moreover, despite organ heterogeneity, the common 73 stress‐induced aging genes are primarily associated with metabolic diseases, cancer, immune responses, and oxidative stress regulation (Supplementary Figure S9), necessitating further research into their roles in UCMS.

We also demonstrated that the UCMS‐induced aging phenotype can be prevented by pharmacological intervention. Our comparative analyses with established anti‐aging agents showed that both BZBS and the NAD^+^ booster NMN effectively restored mitochondrial function and attenuated DNA damage markers, whereas the senolytic ABT‐263 specifically targeted senescent cell clearance without improving metabolic parameters (Supplementary Figure S18a–d). While NMN is well‐established to exert anti‐aging effects primarily through NAD^+^ elevation and Sirtuin activation to improve vascular and neuronal function [60, 61, 62], our data reveals that BZBS consistently outperformed NMN across multiple aging parameters. This superior efficacy may stem from BZBS's ability to simultaneously target multiple nodes of the aging network, including not only Sirtuin activation but also AMPK and PGC1α stimulation, and direct genomic stabilization—effects that NMN cannot achieve through its single‐pathway NAD^+^ boosting mechanism. These findings establish BZBS's unique capacity to concurrently address two fundamental aging processes—cellular senescence through mechanisms similar to ABT‐263 and metabolic decline through pathways akin to NMN—demonstrating a dual therapeutic advantage deriving from its multicomponent nature. Notably, BZBS's anti‐aging effects extend beyond simple additive interactions of its individual components. Systematic combination studies revealed that treatment with Schisantherin A for genomic stability, Schisandrin B for mitochondrial enhancement, and Osthole for oxidative stress alleviation produced significantly greater anti‐senescence effects than any single compound alone (Supplementary Figure S17f–k). This emergent pharmacosynergy appears to operate through multiple interconnected mechanisms, including the concurrent activation of complementary longevity pathways through AMPK activation by Osthole combined with SIRT3 modulation by Schisandrin derivatives and DNA repair potentiation. While our in vitro knockdown experiments using RAW264.7 macrophages (Supplementary Figure S20) demonstrate that BZBS's mitochondrial protective effects strictly require functional AMPK signaling, definitive in vivo validation using myeloid‐specific conditional knockout models remain necessary to fully establish cell‐type‐specific requirements. Additionally, the formulation achieves network‐level stabilization that overcomes biological redundancy in aging processes, while Schisandrin B's mitochondrial improvements synergistically enhance Osthole's redox‐regulatory capacity, collectively creating a self‐reinforcing cycle of cellular rejuvenation. Notably, while C57BL/6 mice were selected for efficacy studies as the optimal model for aging research, we employed rats for pharmacokinetic evaluation due to their larger body size enabling serial blood sampling and reduced inter‐individual variability. Crucially, the conserved pharmacokinetic and pharmacodynamic properties of these bioactive components across rodent species support the translational relevance of our findings.

In summary, our study demonstrates that the UCMS mouse model exhibits accelerated aging features, including system‐wide tissue damage and inflammaging. UCMS triggered immune aging via myeloid expansion, lymphoid depletion, and stress/aging gene upregulation. Both BZBS and NMN improved mitochondrial function and reduced DNA damage, and BZBS showed broad anti‐aging effects. Based on the observation, we propose immune dysfunction may promote oxidative damage through macrophage mtROS. The lung may be a senescence propagation hub, while BZBS's efficacy may arise from multi‐target action (Supplementary Figure S25). However, several limitations warrant consideration—the mouse model, while valuable, cannot fully replicate human aging physiology; the analysis focused on only five major tissues may miss broader systemic interactions; the lack of in vivo validation for candidate genes and the inclusion of only a single anti‐aging drug positive control limits definitive mechanistic interpretation; and the preventive intervention protocol differs from therapeutic clinical scenarios where treatment follows age‐related symptom onset. These considerations highlight important directions for future translational research.

## AI Statement

The authors used DeepSeek‐V4 for language editing purposes only. No AI tools were employed in data analysis, figure preparation, or the generation of scientific content. All authors have reviewed and take full responsibility for the final manuscript.

## Conflicts of Interest

The authors declare no conflicts of interest.

## Supporting information




**Supporting File 1**: exp270235‐sup‐0001‐SuppMat.docx.

**Supporting File 2**: exp270235‐sup‐0002‐SuppMat.xlsx.

**Supporting File 3**: exp270235‐sup‐0003‐SuppMat.xlsx.

**Supporting File 4**: exp270235‐sup‐0004‐SuppMat.xlsx.

**Supporting File 5**: exp270235‐sup‐0005‐FiguresS1‐S25.docx.
