# Supplementary material for: Reversal of Chronic Stress‐Induced Inflammaging by Bazi Bushen Formula Through AMPK‐Sirtuins Axis Activation and Myeloid Reset
Source: Exploration (Beijing). 2026 Oct 11;6(5):70235. doi: 10.1002/exp2.70235 (PMC13654189; doi:10.1002/exp2.70235)
Supplement: Supplementary file 1 [file EXP2-6-70235-s005.docx]

**Table1 The herbal composition of BZBS**

| **#** | **Latin name** | **Chinese pinyin** |
| --- | --- | --- |
| **1** | ***Cuscuta chinensis Lam.*** | **Tu-Si-Zi** |
| **2** | ***Lycium harharum L.*** | **Gou-Qi-Zi** |
| **3** | ***Schisandra chinensis (Turcz.) Baill.*** | **Wu-Wei-Zi** |
| **4** | ***Cnidium monnieri (L.) Cusson*** | **She-Chuang-Zi** |
| **5** | ***Rosa Laevigata Michx.*** | **Jin-Ying-Zi** |
| **6** | ***Rubus chingii Hu*** | **Fu-Pen-Zi** |
| **7** | ***Allium tuberosum Rottler ex Spreng.*** | **Jiu-Cai-Zi** |
| **8** | ***Toosendan fructus*** | **Chuan-Lian-Zi** |
| **9** | ***Epimedium brevicornu Maxim.*** | **Yin-Yang-Huo** |
| **10** | ***Morindae officinalis radix*** | **Ba-Ji-Tian** |
| **11** | ***Cistanche deserticola Ma*** | **Rou-Cong-Rong** |
| **12** | ***Rehmannia root*** | **Di-Huang** |
| **13** | ***Cyathula officinalis K.C.Kuan*** | **Chuan-Niu-Xi** |
| **14** | ***Panax ginseng C.A.Mey.*** | **Ren-Shen** |

**Table 2 QPCR Primer and siRNA sequence List**

| **Genes** | **QPCR Primers** | **Sequences (5′ → 3′)** |
| --- | --- | --- |
| **Nf-κb** | **Forward**  **Reverse** | **GATGTGGTGGAGGACTTGCTGAG**  **GTGCTGCCTTGCTGTTCTTGAG** |
| **Tp53** | **Forward**  **Reverse** | **GGATGTTGCAGAGTTGTTAGAAGGC**  **TTTGAGAAGGGACGGAAGATGACAG** |
| **P21** | **Forward**  **Reverse** | **GCTGGTGGCGTAGGCAAGAG**  **ACCTGCTGTGTCGAGAATATCCAAG** |
| **pl6^INK4a^** | **Forward**  **Reverse** | **AGGGCTTCCTAGACACTCTGGTAG**  **GATACCGCAAATACCGCACGAC** |
| **IL-6** | **Forward**  **Reverse** | **CTTCTTGGGACTGATGCTGGTGAC**  **AGGTCTGTTGGGAGTGGTATCCTC** |
| **TNF-α** | **Forward**  **Reverse** | **GTGCCTATGTCTCAGCCTCT**  **TGGTTTGTGAGTGTGAGGGT** |
| **AMPKα1**  **(mouse)** | **Forward**  **Reverse** | **GTCAAAGCCGACCCAATGATA**  **CGTACACGCAAATAATAGGGGTT** |
| **GADPH**  **(mouse)** | **Forward**  **Reverse** | **GGTTGTCTCCTGCGACTTCA**  **TGGTCCAGGGTTTCTTACTCC** |
| **AMPKα2 (Rat)** | **Forward**  **Reverse** | **TACTGTCACAGGCACATGGT**  **TCCGGTGCTGCATAATTTGG** |
| **GADPH**  **(Rat)** | **Forward**  **Reverse** | **GACATGCCGCCTGGAGAAAC**  **AGCCCAGGATGCCCTTTAGT** |
| **Genes** | **QPCR Primers** | **Sequences (5′ → 3′)** |
| **si- AMPKα1** | **Sequence**  **Antisense sequence** | **CCUGGAUGAAUUAAACCCAdTdT**  **UGGGUUUAAUUCAUCCAGGdTdT** |
| **si- AMPKα2** | **Sequence**  **Antisense sequence** | **GAUGGUGAAUUUCUACGAAdTdT**  **UUCGUAGAAAUUCACCAUCdTdT** |

**Table3. Antibody List**

|  | **Antibody** | **Company** | **Cat no.** | **Dilution** |
| --- | --- | --- | --- | --- |
| **Primary**  **Antibody** | **anti-CD19** | **Abcam** | **ab245235** | **1:250** |
|  | **anti-CD20** | **Abcam** | **ab64088** | **1:100** |
|  | **anti-CD80** | **Abcam** | **ab254579** | **1:500** |
|  | **anti-Mannose Receptor** | **Abcam** | **ab64693** | **1:1000** |
|  | **anti-CDKN2A/pl6INK4a** | **Abcam** | **ab189034** | **1:200** |
|  | **P21** | **Abcam** | **ab188224** | **1：500** |
|  | **AMPK** | **Cell Signaling Technology** | **2532** | **1:1000** |
|  | **P-AMPK** | **Cell Signaling Technology** | **2535** | **1:1000** |
|  | **SIRT1** | **Abcam** | **ab110304** | **1:1000** |
|  | **SIRT3** | **Biodragon** | **RM8142** | **1：2000** |
|  | **SIRT6** | **Biodragon** | **RM8811** | **1：5000** |
|  | **PGC1α** | **Abcam** | **ab191838** | **1：600** |
|  | **anti-Langerin** | **Abcam** | **ab283686** | **1:1000** |
| **Secondary Antibody** | **Goat Anti-Rabbit IgG**  **H&L(Alexa Fluor® 488)** | **Abcam** | **ab150077** | **1:500** |
|  | **Goat Anti-Rabbit IgG**  **H&L(Alexa Fluor® 488)** |  | **ab150083** | **1:500** |

**Table 5. Chemicals and Reagents**

| **Reagent** | **Company** | **Cat no.** | **Lot no.** | |  |
| --- | --- | --- | --- | --- | --- |
| **DMEM** | **gibco** | **11995-073** | | **2891232** | |
| **FBS** | **gibco** | **FSP500** | | **012C-0727A** | |
| **Pancreatic enzymes** | **gibco** | **25200-056** | | **2661765** | |
| **Bispecific antibodies** | **gibco** | **15140-122** | | **2321123** | |
| **DMSO** | **solarbio** | **D8371** | | **34240611007** | |
| **HBSS** | **gibco** | **14025-076** | | **2479861** | |
| **PBS** | **gibco** | **C20012500CP** | | **6123124** | |
| **Serum-free cell cryopreservation solution** | **NCM Biotech** | **C40100** | | **20241026** | |
| **XF Calibrant** | **agilent.** | **100840-000** | | **04123002** | |
| **XF DMEM Medium, pH7.4** | **agilent.** | **103575-100** | | **20623002** | |
| **MTS** | **promega** | **G3581** | | **0000377142** | |
| **MitoSOX™ Red Mitochondrial Superoxide Indicators** | **invitrogen** | **M36008** | | **2615850** | |
| **Mitochondrial membrane potential detection kit** | **beyotime** | **C2006** | | **A077A241203** | |
| **XF Cell Mitochondrial Stress Test Kit** | **agilent.** | **103015-100** | | **18099037** | |
| **Hydrogen peroxide disinfectant** | **Weimin** | **N/A** | | **230702** | |
| **CCCP** | **MCE** | **HY-100941** | | **HY-100941-26** | |
| **ABT-263** | **MCE** | **HY-10087** | | **N/A** | |
| **Dorsomorphin dihydrochloride** | **TargetMol** | **T1977** | | **N/A** | |
| **Cellular AMPK kinase activity photometric quantification assay kit** | **DENMED** | **GMS50140.1.3** | | **1-110902-10** | |
| **RAW264.7 Mouse mononuclear macrophage leukemia cell culture medium** | **zqxzbio** | **ZM0098** | | **DL009826** | |
| **DNA damage detection kits** | **beyotime.** | **C2035S** | | **A133A241226** | |

**Table8** Characterization of prototypes after oral administration of BZBS capsule in rat plasma and brain

| **NO.** | **t_R_ (min)** | **Formula** | **Selected ion** | **Measured mass** | **Calculated mass** | **Mass error /mDa** | **MS fragmentation** | **Identification** | **Source** | **Structure** |
| --- | --- | --- | --- | --- | --- | --- | --- | --- | --- | --- |
| 3 | 4.42 | C_20_H_24_NO_4_ | [M]^+^ | 342.1736 | 342.1705 | 1.7 | 279.1365 | Magnoflorine* | Plasma | Alkaloids |
| 4 | 4.72 | C_9_H_8_O_3_ | [M-H]^-^ | 163.0387 | 163.0395 | -0.8 | 119.0516 | p-Coumaric acid | Plasma | Organic acids |
| 5 | 5.92 | C_14_H_16_O_5_ | [M-H]^-^ | 263.0904 | 263.0919 | -1.5 | 245.0806, 227.0693, 205.0472, **175.0384** | Unknown | Plasma | Others |
| 11 | 7.79 | C_15_H_16_O_4_ | [M+H]^+^ | 261.1125 | 261.1127 | -0.2 | 189.0548, 131.04668 | Auraptenol | Plasma | Coumarins |
| 12 | 8.14 | C_15_H_14_N_2_O_3_ | [M+H]^+^ | 271.1080 | 271.1083 | -0.3 | **225.1034,** 197.1111, 180.0819, **169.0773** | Cuscutamine isomer | Plasma | Alkaloids |
| 16 | 8.60 | C_15_H_14_N_2_O_3_ | [M+H]+ | 271.1086 | 271.1083 | 0.3 | **225.1027,** 197.1089, 180.0819, **169.0766** | Cuscutamine* | Plasma | Alkaloids |
| 20 | 12.76 | C_30_H_48_O_6_ | [M-H]^-^ | 503.3383 | 503.3373 | 1.0 | **N/A** | 19α-Hydroxyasiatic acid* | Plasma | Triterpenes |
| 29 | 15.09 | C_24_H_32_O_7_ | [M+H]^+^ | 433.2234 | 433.2226 | 0.8 | 415.2137, 400.1883, **384.1853, 369.1701,** 338.1540 | Schisandrin* | Plasma/Brain | Lignans |
| 34 | 15.98 | C_15_H_20_O_4_ | [M-H]^-^ | 263.1277 | 263.1283 | -0.6 | 219.1325 | Unknown | Plasma | Others |
| 35 | 16.06 | C_28_H_34_O_10_ | [M+H]^+^ | 531.2208 | 531.2230 | -2.2 | 401.1599, 383.1519, 352.1304, **337.1071,** 326.1162 | Gomisin D | Plasma | Lignans |
| 36 | 16.93 | C_16_H_14_O_4_ | [M+H]^+^ | 271.0955 | 271.0970 | -1.5 | 203.0341 | Imperatorin * | Plasma | Coumarins |
| 37 | 17.92 | C_15_H_16_O_3_ | [M+H]^+^ | 245.1178 | 245.1178 | 0.0 | **189.0587, 131.0493,** 103.0540 | Osthole* | Plasma/Brain | Coumarins |
| 38 | 18.18 | C_28_H_36_O_8_ | [M+Na]^+^ | 523.2256 | 523.2308 | -5.2 | 401.1959 | Tigloylgomisin H | Plasma | Lignans |
| 39 | 18.76 | C_24_H_30_O_7_ | [M+H]^+^ | 431.2076 | 431.2070 | 0.6 | 401.1908, 389.1596, 372.1597, **356.1639,** 341.1394 | Schisanlignone A or Schisantherin S | Plasma | Lignans |
| 40 | 19.59 | C_23_H_30_O_6_ | [M+H]^+^ | 403.2122 | 403.2121 | 0.1 | 372.1924, 340.1639 | Schisanhenol isomer | Plasma | Lignans |
| 41 | 19.67 | C_30_H_32_O_9_ | [M+H]^+^ | 537.2117 | 537.2125 | -0.8 | 437.1589, 415.1733, 385.1694, **371.1485, 340.1328** | Gomisin G | Plasma | Lignans |
| 42 | 19.91 | C_30_H_32_O_9_ | [M+Na]^+^ | 559.1926 | 559.1944 | -1.8 | 415.1784, **371.1482,** 356.1307, 342.1095, **325.1071** | Schisantherin A* | Plasma | Lignans |
| 43 | 20.06 | C_28_H_34_O_9_ | [M+Na]^+^ | 537.2108 | 537.2101 | 0.7 | 415.1747, 385.1644, **371.1496, 340.1306,** 325.1075 | Schisantherin B* | Plasma | Lignans |
| 45 | 20.50 | C_23_H_30_O_6_ | [M+H]^+^ | 403.2111 | 403.2121 | -1.0 | 340.1691 | Schisanhenol* | Plasma | Lignans |
| 50 | 22.04 | C_24_H_32_O_6_ | [M+H]^+^ | 417.2283 | 417.2277 | -0.2 | 402.2062, 386.2104, 347.1459, 316.1277, 301.1064 | Deoxyschisandrin* | Plasma/Brain | Lignans |
| 51 | 22.69 | C_23_H_28_O_6_ | [M+H]^+^ | 401.1965 | 401.1964 | 0.1 | 386.1712, 370.1792, 354.1409, **331.1208,** 300.1006 | Schisandrin B isomer | Plasma/Brain | Lignans |
| 52 | 22.86 | C_23_H_28_O_6_ | [M+H]^+^ | 401.1967 | 401.1964 | 0.1 | 386.1750, 370.1800, 340.1686, **300.0988, 285.0775** | Schisandrin B* | Plasma/Brain | Lignans |
| 53 | 23.47 | C_28_H_34_O_8_ | [M+Na]^+^ | 521.2147 | 521.2151 | -0.4 | 399.1790, 368.1621, 337.1438 | Angeloylgomisin R or Angeloylgomisin O | Plasma | Lignans |

*: Comparison with reference standards
