# Supplementary material for: Reversal of Chronic Stress‐Induced Inflammaging by Bazi Bushen Formula Through AMPK‐Sirtuins Axis Activation and Myeloid Reset
Source: Exploration (Beijing). 2026 Oct 11;6(5):70235. doi: 10.1002/exp2.70235 (PMC13654189; doi:10.1002/exp2.70235)
Supplement: Supplementary file 2 [file EXP2-6-70235-s003.docx]

**Supplementary Figure 1. Physiological and behavioral validation of baseline aging status and UCMS model efficacy. (a)** Stressed mice exhibited significantly elevated serum aldosterone levels compared to non-stressed controls, confirming hypothalamic-pituitary-adrenal (HPA) axis activation (n=3). **(b)** Body weight loss was observed in UCMS-exposed mice throughout the stress protocol, supporting the successful induction of chronic stress (n=3). **(c)** Representative photographs showing physical appearance of 11-month-old (middle-aged) and 4-month-old (young control) mice. **(d)** No significant difference in FI was observed between groups (n=6). **(e)** Rotarod performance latency remained comparable between middle-aged and young mice (n=6). **(f)** Forelimb grip strength measurements demonstrated no age-associated decline (n=6). **(g-i)** Barnes maze evaluations including representative path tracings (g), total time (h) (n=6), and total movement distance (i) (n=6) showed no cognitive impairment in middle-aged mice compared to young controls. Data are presented as mean ± SEM. Statistical significance was analyzed by unpaired two-tailed Student's t-test (*p < 0.05, **p < 0.01, ***p < 0.001).

**Supplementary Figure 2.** **Complementary histological evidence of stress-driven multi-organ degeneration**. **(a)** The Masson staining of mouse skin. **(b)** The statistics of collagen content in the mouse skin (n=3). **(c)** The H&E staining of mouse kidney. **(d)** The H&E staining of mouse lungs. White arrows indicate inflammatory cell infiltration. Data presented as mean ± SD. Statistical significance determined by one-way ANOVA with Tukey post hoc (multi-group, *p<0.05, **p<0.01, ***p<0.001).

**Supplementary Figure 3.** **Therapeutic modulation of immune polarization. (a-f)** The expression levels of CD207^+^, CD19^+^, CD20^+^, CD80^+^, and CD206^+^ immune cells in mouse skin. n=3-6 for the biologically independent samples . **(g-o)** The staining and statistical analysis of CD80^+^, CD206^+^, and CD19^+^ immune cells in mouse (g, j) intestines, (h, k) adipose, (i, l) brains and (m-o) hearts. n=3-6 for the biologically independent samples. Data presented as mean ± SD. Statistical significance determined by one-way ANOVA with Tukey post hoc (multi-group, *p<0.05, **p<0.01, ***p<0.001).

**Supplementary Figure 4.** **Chronic stress disrupts B cell differentiation and tissue distribution. (a)** The UMAP graph shows that BCs from the five types of samples can be further divided into eight subtypes (clusters). **(b)** The heatmap of marker genes expression in each cluster. **(c)** AUCell scores for BC-related biological processes from the GO database. The scores are scaled row-wise. Red indicates more active related functions, while blue indicates less expression of related genes. **(d)** The enriched GO terms/pathways (TOP20) for the BC_C2 marker gene. **(e)** The monocle3 analysis shows the differentiation trajectory among BC subtypes. The color gradient from dark to light represents the start to the end of pseudotime during differentiation. **(f)** CellRank analysis based on RNA velocity indicates the direction of differentiation among BC sub-types, as suggested by the arrows with streamlines. **(g, h)** The distribution of BC subtypes among the three groups varies. The Model group has a lower overall number of BCs. Different colors represent different BC subtypes. **(i)** Tissue-specific subcluster distribution.

**Supplementary Figure 5.** **Macrophage pro-inflammatory polarization and lung-specific dynamics. (a,b)** UMAP clustering of 11 macrophage subgroups and their marker genes. **(c)** AUCell scores for macrophage -related biological processes from the GO database. **(d, e)** The distribution of macrophage subtypes among the three groups varies. **(f)** Tissue-specific macrophage subcluster distribution.

**Supplementary Figure 6.** **Stress-Driven** **Neutrophil Priming and Pulmonary Compartmentalization. (a, b)** UMAP clustering of 6 neutrophil subgroups and their marker genes. **(c)** AUCell scores for neutrophil-related biological processes from the GO database. **(d, e)** Pseudotime/RNA velocity analysis showing the direction of differentiation among neutrophil subcluster. **(f, g)** The distribution of neutrophil subcluster among the three groups varies. **(h)** Tissue-specific neutrophil subcluster distribution. **(i)** Summary of changes in lung immune cells under stress.

**Supplementary Figure 7.** **Inflammaging and communication dynamics. (a, b)** Crosstalk between myeloid and lymphoid immune cells and somatic cells in intestine and lung.

**Supplementary Figure 8.** **Molecular validation of inflammaging phenotypes. (a-n)** qPCR analysis of (a-g) inflammatory factors (*NF-κB*, *TNF-α*, and *IL-6*) and (h-n) senescence markers (*P16^INK4a^*, *P21*, and *P53*) in tissues of control, model and BZBS groups (n=3). **(o, p)** Brain Iba-1^+^ microglia staining. Scale bars: 40-80 μm. n=3 for the biologically independent samples (n=3-5 for p). Data presented as mean ± SD. Statistical significance determined by one-way ANOVA with Tukey post hoc (multi-group, *p<0.05, **p<0.01, ***p<0.001).

**Supplementary Figure 9.** **Shared and unique stress-senescence gene signatures.** **(a)** Venn diagram of common stress-DEGs across five tissues. **(b)** Heatmap of stress-DEGs across five tissues.

**Supplementary Figure 10.** **BZBS reverses transcriptional stress burden. (a)** Tissue AUCell stress scores upon BZBS treatments. **(b)** Correlation analysis between inflammation response scores and stress level scores for various cell types. **(c)** Augur score of stress-DEGs in each cell type of tissues upon BZBS treatments. **(d)** Cell AUCell stress scores upon BZBS treatments.

**Supplementary Figure 11.** **BZBS reverses cell-type-specific aging gene expression. (a-c)** Proportion of aging-related differentially expressed genes (DEGs) reversed by BZBS across tissues. **(d)** Tissue-specific aging scores quantified by AUCell following BZBS treatment. **(e, f)** Proportion of aging-related DEGs reversed by BZBS across different cell types. **(g)** Cell-type-specific aging scores quantified by AUCell upon BZBS treatment.

**Supplementary Figure 12.** **BZBS alleviates protein and lipid peroxidation in multiple tissues. (a)** Tissue-specific lipid peroxide levels across experimental conditions (n=4-6). **(b)** Tissue-specific protein carbonylation levels across experimental conditions (n=5-6). **(c)** Tissue-specific SOD activity across experimental conditions (n=5-6). **(d)** Tissue-specific GSH levels across experimental conditions (n=6). Data presented as mean ± SD. Statistical significance determined by one-way ANOVA with Tukey post hoc (multi-group, *p<0.05, **p<0.01, ***p<0.001).

**Supplementary Figure 13.** **BZBS attenuates mtROS buildup in aging kidney-associated macrophages. (a-c)** Renal immune fluorescence staining showing elevated mtROS in infiltrating macrophages (myeloid cells) in aging-model mice and attenuation by BZBS treatment, linking immune cell dysfunction to tissue oxidative stress (n=5). Data presented as mean ± SD. Statistical significance determined by one-way ANOVA with Tukey post hoc (multi-group, *p<0.05, **p<0.01, ***p<0.001).

**Supplementary Figure 14.** **Epigenetic alterations in stress-induced aging mice and rescue by BZBS treatment. (a)** Comparative DNA methylation levels across functional genomic regions (promoters, exons, introns, intergenic) in control, model, and BZBS-treated groups (upper). Methylation landscape analysis at gene regulatory regions (TSS ± 2kb, including 5' and 3' flanking sequences) (middle). CpG island (CGI) methylation profiles showing hypermethylation in model animals (lower). **(b)** Epigenetic age acceleration assessed using liver DNA methylation clocks, with stress-induced aging mice exhibiting elevated methylation age relative to chronological age - a phenotype attenuated by BZBS therapy (n=5). Data are presented as mean ± SEM. Statistical significance was analyzed by unpaired two-tailed Student's t-test (*p < 0.05, **p < 0.01, ***p < 0.001).

**Supplementary Figure 15.** **UPLC/Q-TOF-MS analysis of BZBS. (a)** Extracted ion chromatograms of prototype compounds in drug-containing rat plasma, as identified by UPLC-QTOF-MS. **(b, c)** Extracted ion chromatograms of prototype compounds in drug-containing rat brain tissue, as identified by UPLC-QTOF-MS.

**Supplementary Figure 16.** **Compounds in BZBS alleviate mitochondrial stress. (a, b)** Mitochondrial ROS generation induced by varying concentrations of CCCP (n=5-6). **(c)** Cytotoxic effects of compounds on normal astrocytes. The dotted line indicates 80% cell viability (n=3-6). **(d, e)** Effects of compounds on reducing mitochondrial ROS in astrocytes stimulated by hydrogen peroxide (n=5-6 for d, n=6 for e). Data presented as mean ± SEM. Statistical significance determined by one-way ANOVA with Tukey post hoc test. Asterisks (*) indicate treatment vs. vehicle control (*p < 0.05, **p < 0.01, ***p < 0.001). Hashtags (#) indicate model group vs. untreated control (#p < 0.05, ##p < 0.01, ###p < 0.001).

**Supplementary Figure 17.** **Synergistic anti-senescence effects of compound combination in irradiation-induced senescence models. (a, b)** γH2AX immunostaining and quantification demonstrating superior DNA damage attenuation by the combined treatment compared to individual compounds (n=5). (c, d) P21 staining and quantification revealing enhanced suppression of cellular senescence with triple-compound therapy versus monotherapies (n=5). Quantitative data presented as mean ± SD. Statistical analysis by one-way ANOVA with Tukey's post-hoc test (multi-group, *p<0.05, **p<0.01, **p<0.001).

**Supplementary Figure 18.** **Mechanistic benchmarking of BZBS against established anti-aging compounds in irradiation-induced senescence models. (a, b)** Mitochondrial ROS levels after treating with BZBS, NMN, and ABT-263 (n=5). **(c, d)** γH2AX quantification demonstrating DNA damage repair by BZBS, NMN, and ABT-263 (n=5). Quantitative data presented as mean ± SD**.** Statistical significance was analyzed by one-way ANOVA with Tukey's post-hoc test (*p < 0.05, **p < 0.01, ***p < 0.001).

**Supplementary Figure 19. Network pharmacology analysis of BZBS active substances targeting mitochondria and epigenetic regulation. (a)** Venn diagram illustrating the overlap between potential compound targets epigenetic regulation-related and oxidative stress-related targets. **(b)** Network of potential targets associated with active compounds involved in epigenetic and oxidative stress regulation. **(c, d)** Protein-protein interaction (PPI) network of potential share targets. **(e)** Schematic representation of potential targets linked to key biological processes in oxidative stress and epigenetic regulation.

**Supplementary Figure 20. AMPKαmediates BZBS-induced mitochondrial and genomic protection in senescent cells. (a)** qPCR (n=3) and **(b, c)** Western blot analysis (n=4 for b, n=5 for c) confirm efficient AMPKα1/2 knockdown in RAW264.7 macrophages and CTX cells following siRNA transfection. **(d, e)** mtROS staining demonstrates that BZBS treatment fails to restore stress-induced mitochondrial dysfunction in AMPKα1/2-deficient cells (n=5). **(f, g)** γH2AX immunofluorescence shows persistent DNA damage in knockdown cells despite BZBS administration (n=5). Quantitative data presented as mean ± SD**.** Statistical significance was analyzed by one-way ANOVA with Tukey's post-hoc test (*p < 0.05, **p < 0.01, ***p < 0.001).

**Supplementary Figure 21. Virtual docking of BZBS active substances with oxidative stress and epigenetic regulation targets. (a-d)** Molecular docking analysis of active compounds with AMPK. **(e, f)** Molecular docking analysis of active compounds with SIRT1. **(g, h)** Molecular docking analysis of active compounds with SIRT3. **(i)** Molecular docking analysis of active compound with SIRT6.

**Supplementary Figure 22. Target engagement validation of BZBS active components via CETSA. (a-d)** Thermal stability profiles of endogenous (a) AMPKα, (b) SIRT1, (c) SIRT6, and (d) SIRT3 in HEK293 cells treated with vehicle or 20 μM of individual BZBS components. Quantitative data presented as mean ± SD, with n=3 biological replicates per group. Statistical analysis by one-way ANOVA with Tukey's post-hoc test (*p < 0.05, **p < 0.01, ***p < 0.001).

**Supplementary Figure 23. Full western blot images supporting all main and supplementary figures.** Full western blot images supporting all main and supplementary figures (excluding CETSA experiments) are provided.

**Supplementary Figure 24. Full western blot images from CETSA.** Full western blot images from CETSA assays (Supplementary Figure 22) are provided for completeness, demonstrating the thermal stability profiles of target proteins.

**Supplementary Figure 25. Chronic Stress Triggers Myeloid-Lymphoid Imbalance and Multi-Organ Inflammaging: Reversal by Multi-Targeting Therapeutic Strategy.** Chronic stressors trigger systemic immune remodeling characterized by myeloid expansion and lymphoid suppression. This immune imbalance propagates tissue pathology via pro-inflammatory signaling and senescence amplification, with the lung and kidney identified as the most vulnerable organs. Such cascading alterations accelerate healthspan decline and lifespan shortening. BZBS combats these effects by synergistically targeting mitochondrial dysfunction, genomic instability, and epigenetic dysregulation.
